# Mean-Field Limits: From Particle Descriptions to Macroscopic Equations

**DOI:** 10.1007/s00205-021-01676-x

**Published:** 2021-06-01

**Authors:** José A. Carrillo, Young-Pil Choi

**Affiliations:** 1 Department of Mathematics Mathematical Institute, University of Oxford, Oxford, OX2 6GG UK; 2 Department of Mathematics, Yonsei University, 50 Yonsei-Ro, Seodaemun-Gu, Seoul, 03722 Republic of Korea

## Abstract

We rigorously derive pressureless Euler-type equations with nonlocal dissipative terms in velocity and aggregation equations with nonlocal velocity fields from Newton-type particle descriptions of swarming models with alignment interactions. Crucially, we make use of a discrete version of a modulated kinetic energy together with the bounded Lipschitz distance for measures in order to control terms in its time derivative due to the nonlocal interactions.

## Introduction

In this work, we analyse the evolution of an indistinguishable *N*-point particle system given by

1.1 $$\begin{aligned} \begin{aligned} \dot{x}_i&= v_i, \quad i=1,\dots ,N, \quad t > 0,\\ \varepsilon _N \dot{v}_i&=-\gamma v_i - \nabla _x V(x_i) - \frac{1}{N} \sum _{j=1}^N \nabla _x W(x_i - x_j)+ \frac{1}{N} \sum _{j=1}^N \psi (x_i - x_j)(v_j - v_i) \end{aligned} \end{aligned}$$

subject to the initial data

1.2 $$\begin{aligned} (x_i,v_i)(0) =: (x_i(0), v_i(0)), \quad i =1,\dots ,N. \end{aligned}$$

Here $x_i = x_i(t) \in {\mathbb {R}}^d$ and $v_i = v_i(t) \in {\mathbb {R}}^d$ denote the position and velocity of *i*-particle at time *t*, respectively. The coefficient $\gamma \geqq 0$ represents the strength of linear damping in velocity, $\varepsilon _N>0$ the strength of inertia, $V:{\mathbb {R}}^d \rightarrow {\mathbb {R}}_+$ and $W : {\mathbb {R}}^d \rightarrow {\mathbb {R}}$ stand for the confinement and interaction potentials, respectively. $\psi : {\mathbb {R}}^d \rightarrow {\mathbb {R}}_+$ is a communication weight function. Throughout this paper, we assume that *W* and $\psi$ satisfy $W(x) = W(-x)$ and $\psi (x) = \psi (-x)$ for $x \in {\mathbb {R}}^d$. They include basic particle models for collective behaviors, see [12, 20, 25, 34, 36, 46, 47, 63] and the references therein.

Our main goal is to derive the macroscopic collective models rigorously governing the evolution of the particle system (1.1) as the number of particles goes to infinity. On one hand, we will derive hydrodynamic Euler-alignment models given by

1.3 $$\begin{aligned} \begin{aligned}&\partial _t \rho + \nabla _x \cdot (\rho u) = 0,\\&\partial _t (\rho u) + \nabla _x \cdot (\rho u \otimes u) = -\gamma \rho u - \rho \nabla _x V - \rho \nabla _x W \star \rho \\&\quad + \rho \int _{{\mathbb {R}}^d} \psi (x-y) (u(y) - u(x))\,\rho (y)\,\mathrm{d}y \end{aligned} \end{aligned}$$

in the mean-field limit: when initial particles are close to a monokinetic distribution $\rho _0(x) \delta _{u_0(x)}(v)$ in certain sense and $\varepsilon _N=O(1)$ as $N\rightarrow \infty$. On the other hand, we will show that the particle system can be described by aggregation equations of the form

1.4 $$\begin{aligned} \partial _t {\bar{\rho }} + \nabla _x \cdot ({\bar{\rho }} {\bar{u}}) = 0, \end{aligned}$$

where

1.5 $$\begin{aligned} \gamma {\bar{\rho }} {\bar{u}} = - {\bar{\rho }} \nabla _x V - {\bar{\rho }} \nabla _x W \star {\bar{\rho }} +{\bar{\rho }} \int _{{\mathbb {R}}^d}\psi (x-y)({\bar{u}}(y) - {\bar{u}}(x)) {\bar{\rho }}(y)\,\mathrm{d}y \end{aligned}$$

in the combined mean-field/small inertia limit when initial particles are close to a monokinetic distribution $\rho _0(x) \delta _{u_0(x)}(v)$, $\gamma >0$ and $\varepsilon _N \rightarrow 0$ as $N \rightarrow \infty$. For simplicity of notations when dealing with the mean-field limit, we will take $\varepsilon _N=1$ in the sequel.

### Mean-field limits: from particles to continuum

As the number of particles *N* tends to infinity, microscopic descriptions given by the particle system (1.1) become more and more computationally unbearable. Reducing the complexity of the system is of paramount importance in any practical application. The classical multiscale strategy in kinetic modelling is to introduce the number density function $f = f(x,v,t)$ in phase space $(x,v) \in {\mathbb {R}}^d \times {\mathbb {R}}^d$ at time $t \in {\mathbb {R}}_+$ and study the time evolution of that density function. Then at the formal level, we can derive the following Vlasov-type equation from the particle system (1.1) as $N \rightarrow \infty$:

1.6 $$\begin{aligned} \partial _t f + v \cdot \nabla _x f - \nabla _v \cdot \left( (\gamma v + \nabla _x V + \nabla _x W \star \rho _f )f\right) + \nabla _v \cdot (F_a(f)f) =0,\nonumber \\ \end{aligned}$$

where $\rho _f = \rho _f(x,t)$ is the local particle density and $F_a(f) = F_a(f)(x,v,t)$ represents a nonlocal velocity alignment force given by

$$\begin{aligned} \rho _f(x,t) := \int _{{\mathbb {R}}^d}f(x,v,t)\,\mathrm{d}v \end{aligned}$$

and

$$\begin{aligned} F_a(f)(x,v,t) := \iint _{{\mathbb {R}}^d \times {\mathbb {R}}^d}\psi (x-y)(w-v)f(y,w,t)\,\mathrm{d}y\,\mathrm{d}w, \end{aligned}$$

respectively. Let us briefly recall the reader the basic formalism leading to the kinetic equation (1.6) as the limiting system of (1.1). We first define the empirical measure $\mu ^N$ associated to a solution to the particle system (1.1), that is,

$$\begin{aligned} \mu ^N_t(x,v) := \frac{1}{N} \sum _{i=1}^N \delta _{(x_i(t), v_i(t))}. \end{aligned}$$

As long as there exists a solution to (1.1), the empirical measure $\mu ^N$ satisfies (1.6) in the sense of distributions. To be more specific, for any $\varphi \in {\mathcal {C}}^1_0({\mathbb {R}}^d \times {\mathbb {R}}^d)$, we get

1.7 $$\begin{aligned} \begin{aligned}&\frac{\mathrm{d}}{\mathrm{d}t}\iint _{{\mathbb {R}}^d \times {\mathbb {R}}^d}\varphi (x,v)\,\mu ^N_t(\mathrm{d}x\mathrm{d}v)= \frac{\mathrm{d}}{\mathrm{d}t} \frac{1}{N} \sum _{i=1}^N \varphi (x_i(t), v_i(t))\\&\quad = \frac{1}{N} \sum _{i=1}^N \left( \nabla _x \varphi (x_i(t), v_i(t)) \cdot v_i(t) + \nabla _v \varphi (x_i(t), v_i(t)) \cdot \dot{v_i}(t) \right) . \end{aligned} \end{aligned}$$

Notice that the particle velocity can also be rewritten in terms of the empirical measure $\mu ^N$ as

$$\begin{aligned} \dot{v_i}(t)= & {} -\gamma v_i - \nabla _x V(x_i) - \iint _{{\mathbb {R}}^d \times {\mathbb {R}}^d}\nabla _x W(x_i - y)\,\mu ^N_t(\mathrm{d}y\mathrm{d}w) \\&+ \iint _{{\mathbb {R}}^d \times {\mathbb {R}}^d}\psi (x_i - y)(w-v_i)\,\mu ^N_t(\mathrm{d}y\mathrm{d}w). \end{aligned}$$

This implies that the right-hand side of (1.7) can also be written in terms of the empirical measure $\mu ^N$ as

$$\begin{aligned} \begin{aligned}&\frac{\mathrm{d}}{\mathrm{d}t}\iint _{{\mathbb {R}}^d \times {\mathbb {R}}^d}\varphi (x,v) \, \mu ^N_t(\mathrm{d}x\mathrm{d}v) = \iint _{{\mathbb {R}}^d \times {\mathbb {R}}^d}\nabla _x \varphi (x,v)\,\mu ^N_t(\mathrm{d}x\mathrm{d}v)\\&\quad -\iint _{{\mathbb {R}}^d \times {\mathbb {R}}^d}\nabla _v \varphi (x,v) \cdot \left( \gamma v + \nabla _x V(x) +\iint _{{\mathbb {R}}^d \times {\mathbb {R}}^d}\nabla _x W(x - y)\,\mu ^N_t(dydw) \right) \mu ^N_t(\mathrm{d}x\mathrm{d}v)\\&\quad + \iint _{{\mathbb {R}}^d \times {\mathbb {R}}^d}\nabla _v \varphi (x, v) \cdot \left( \iint _{{\mathbb {R}}^d \times {\mathbb {R}}^d}\psi (x - y) (w-v)\,\mu ^N_t(\mathrm{d}y\mathrm{d}w)\right) \mu ^N_t(\mathrm{d}x\mathrm{d}v). \end{aligned} \end{aligned}$$

This concludes that $\mu ^N$ is a solution to (1.6) in the sense of distributions as long as particle paths are well defined. In fact, if the interaction potential *W* and the communication weight function $\psi$ in the classical Cucker–Smale alignment model are regular enough, for instance, bounded Lipschitz regularity, then the global-in-time existence of measure-valued solutions can be obtained by establishing a weak-weak stability estimate for the empirical measure, see [46, Section 5] for more details. The mean-field limit has attracted lots of attention in the last years in different settings depending on the regularity of the involved potentials *V*, *W* and communication function $\psi$. Different approaches to the derivation of the Vlasov-like kinetic equations with alignments/interaction terms or the aggregation equations have been taken leading to a very lively interaction between different communities of researchers in analysis and probability. We refer to [3, 4, 10, 20, 30, 31, 35, 44, 47, 50, 54–56, 64, 67] for the classical references and non-Lipschitz regularity velocity fields in kinetic cases, to [48, 49] for very related incompressible fluid problems, and to [7, 9, 16, 17, 37, 43, 45, 51, 52, 61, 63, 65, 66] for results with more emphasis on the singular interaction kernels both at the kinetic and the aggregation-diffusion equation cases.

### Local balanced laws, the mono-kinetic ansatz, and the small inertia limit

The classical procedure in kinetic theory of deriving equations for the first 3 moments of the distribution function *f* leads to the standard problem of how to close the moment system since the equation for the second moment will depend on higher order moments. Suitable closure assumptions are not known so far even in cases where noise/diffusion is added to the system. However, at the formal level, we can take into account the mono-kinetic ansatz for *f*, as done in [18, 21], leading to

1.8 $$\begin{aligned} f (x,v,t) \simeq \rho (x,t) \delta _{u(x,t)}(v)\,, \end{aligned}$$

where $\rho$ and *u* are the macroscopic density and the mean velocity of particles, that is, the first two moments of *f* in velocity variable

$$\begin{aligned} \rho := \int _{{\mathbb {R}}^d}f\,dv \qquad \text{ and } \qquad \rho u := \int _{{\mathbb {R}}^d}vf\,\mathrm{d}v. \end{aligned}$$

It is standard to check that the strain tensor and heat flux become zero and the moment system closes becoming the pressureless Euler equations with nonlocal interaction forces (1.3):

1.9 $$\begin{aligned} \begin{aligned}&\partial _t \rho + \nabla _x \cdot (\rho u) =0, \quad (x,t) \in {\mathbb {R}}^d \times {\mathbb {R}}_+,\\&\quad \partial _t u + u \cdot \nabla _x u = - \gamma u - \nabla _x V - \nabla _x W \star \rho + \int _{{\mathbb {R}}^d} \psi (x-y)(u(y) - u(x))\rho (y)\,\mathrm{d}y, \end{aligned} \end{aligned}$$

and

$$\begin{aligned}&\partial _t \frac{|u|^2}{2} + u\cdot \nabla _x \frac{|u|^2}{2}= -\gamma |u|^2 - u \cdot \nabla _x V - u \cdot \nabla _x W \star \rho \\&\quad + \int _{{\mathbb {R}}^d}\psi (x-y) \left( u(x) \cdot u(y) - |u(x)|^2 \right) \rho (y)\,\mathrm{d}y \end{aligned}$$

on the support of $\rho$. The last equation coming from the closed equation on the evolution of the second moment is redundant but it gives a nice information about the total energy of the system. Although the monokinetic assumption is not fully rigorously justified and it does not have a direct physical motivation, it is observed by particle simulations that the derived hydrodynamic system shares some qualitative behavior with the particle system, see [12, 18, 20–22, 33]. Note that (1.3) conserves only the total mass in time in this generality. However, the total free energy is dissipated due to the linear damping and the velocity alignment force as pointed out in [19] for weak solutions of this system. The hydrodynamic system (1.9) has a rich variety of phenomena compared to the plain pressureless Euler system. This fact is due to the competition between attraction/repulsion and alignment leading to sharp thresholds for the global existence of strong solutions versus finite time blow-up and decay to equilibrium, see [13–15, 26, 63, 68]. We emphasize that the additional alignment, linear damping and attraction/repulsion terms can promote the existence of global solutions depending on the intial data. We will show that these hydrodynamical solutions can be obtained directly from particle descriptions as long as they exist, so their physical relevance is dictated by the time of existence of these solutions.

It is worth noticing as in [18] that the mono-kinetic ansatz for *f* is a measure-valued solution of the kinetic equation (1.6). More precisely, one can show that $\rho (x,t) \delta _{u(x,t)}(v)$ is a solution to the kinetic equation (1.6) in the sense of distributions as long as $(\rho ,u)(x,t)$ is a strong solution to the hydrodynamic equations (1.3). Indeed, for any $\varphi \in {\mathcal {C}}^1_0({\mathbb {R}}^d \times {\mathbb {R}}^d)$, we obtain

$$\begin{aligned} \begin{aligned}&\frac{\mathrm{d}}{\mathrm{d}t}\iint _{{\mathbb {R}}^d \times {\mathbb {R}}^d}\varphi (x,v) \rho (x,t) \,\delta _{u(x,t)}(\mathrm{d}v)\,\mathrm{d}x\\&\quad = \frac{\mathrm{d}}{\mathrm{d}t}\int _{{\mathbb {R}}^d}\varphi (x,u(x,t)) \rho (x,t) \,\mathrm{d}x = \int _{{\mathbb {R}}^d}\varphi (x,u(x,t)) \partial _t \rho \,\mathrm{d}x \\&\qquad + \int _{{\mathbb {R}}^d}(\nabla _v \varphi ) (x, u(x,t)) \cdot (\partial _t u) \rho \,\mathrm{d}x =: I_1 + I_2. \end{aligned} \end{aligned}$$

Using the continuity equation in (1.3), $I_1$ can be easily rewritten as

$$\begin{aligned} \begin{aligned} I_1&= \int _{{\mathbb {R}}^d}\nabla _x (\varphi (x, u(x,t))) \cdot (\rho u)\,\mathrm{d}x \\&= \iint _{{\mathbb {R}}^d \times {\mathbb {R}}^d}(\nabla _x \varphi )(x,v)\cdot (\rho v) \delta _{u(x,t)}(\mathrm{d}v)\,\mathrm{d}x+ \int _{{\mathbb {R}}^d}(\nabla _v \varphi )(x,u(x,t)) \cdot \rho (u \cdot \nabla _x) u\,\mathrm{d}x. \end{aligned} \end{aligned}$$

By multiplying the velocity equation in (1.3) by $\rho$ and using $(\nabla _v \varphi ) (x, u(x,t))$ as a test function to the resulting equation yields

$$\begin{aligned} \begin{aligned} I_2&= - \int _{{\mathbb {R}}^d}(\nabla _v \varphi ) (x, u(x,t)) \cdot (\partial _t u) \rho \,\mathrm{d}x \\&\quad - \int _{{\mathbb {R}}^d}(\nabla _v \varphi ) (x, u(x,t)) \cdot \left( \gamma u + \nabla _x V + \nabla _x W \star \rho \right) \rho \,\mathrm{d}x\\&\quad + \iint _{{\mathbb {R}}^d \times {\mathbb {R}}^d}(\nabla _v \varphi ) (x, u(x,t)) \cdot (u(y) - u(x)) \psi (x-y)\rho (x)\rho (y)\,\mathrm{d}x\mathrm{d}y. \end{aligned} \end{aligned}$$

Then similarly as before, we can rewrite the second and third terms on the right hand side of the equality by using the mono-kinetic ansatz (1.8). This implies

$$\begin{aligned} \begin{aligned} I_2&= - \int _{{\mathbb {R}}^d}(\nabla _v \varphi ) (x, u(x,t)) \cdot (\partial _t u) \rho \,\mathrm{d}x \\&\quad - \iint _{{\mathbb {R}}^d \times {\mathbb {R}}^d}(\nabla _v \varphi ) (x, v) \cdot \left( \gamma v + \nabla _x V + \nabla _x W \star \rho \right) \rho \delta _{u(x,t)}(\mathrm{d}v)\,\mathrm{d}x\\&\quad + \iiiint _{{\mathbb {R}}^d \times {\mathbb {R}}^d \times {\mathbb {R}}^d \times {\mathbb {R}}^d} (\nabla _v \varphi ) (x, v) \\&\quad \cdot (w - v) \psi (x-y)\rho (x)\delta _{u(x,y)}(\mathrm{d}v)\rho (y)\delta _{u(y,t)}(\mathrm{d}w)\,\mathrm{d}x\mathrm{d}y. \end{aligned} \end{aligned}$$

Combining all of the above estimates yields

$$\begin{aligned} \begin{aligned}&\frac{\mathrm{d}}{\mathrm{d}t}\iint _{{\mathbb {R}}^d \times {\mathbb {R}}^d}\varphi (x,v) \rho (x,t) \delta _{u(x,t)}(\mathrm{d}v)\,\mathrm{d}x= \iint _{{\mathbb {R}}^d \times {\mathbb {R}}^d}((\nabla _x \varphi )(x,v)\cdot v) \rho \delta _{u(x,t)}(\mathrm{d}v)\,\mathrm{d}x\\&\quad - \iint _{{\mathbb {R}}^d \times {\mathbb {R}}^d}(\nabla _v \varphi ) (x, v) \cdot \left( \gamma v + \nabla _x V + \nabla _x W \star \rho \right) \rho \delta _{u(x,t)}(\mathrm{d}v)\,\mathrm{d}x\\&\quad +\iiiint _{{\mathbb {R}}^d \times {\mathbb {R}}^d \times {\mathbb {R}}^d \times {\mathbb {R}}^d} (\nabla _v \varphi ) (x, v) \\&\quad \cdot (w - v) \psi (x-y)\rho (x)\delta _{u(x,y)}(dv)\rho (y)\delta _{u(y,t)}(\mathrm{d}w)\,\mathrm{d}x\mathrm{d}y. \end{aligned} \end{aligned}$$

This shows that $\rho (x,t) \delta _{u(x,t)}(v)$ satisfies the kinetic equation (1.6) in the sense of distributions.

Finally, we will be also dealing with the small inertia limit for both the kinetic equation (1.6) and the hydrodynamic system (1.3) combined with the mean field limit. In the small inertia asymptotic limit, we want to describe the behavior of the scaled kinetic equation

1.10 $$\begin{aligned} \varepsilon (\partial _t f + v \cdot \nabla _x f) - \nabla _v \cdot \left( (\gamma v + \nabla _x V + \nabla _x W \star \rho _f )f\right) + \nabla _v \cdot (F_a(f)f) =0,\nonumber \\ \end{aligned}$$

and the scaled hydrodynamic system

1.11 $$\begin{aligned} \begin{aligned}&\partial _t \rho + \nabla _x \cdot (\rho u) = 0,\\&\quad \varepsilon (\partial _t (\rho u) + \nabla _x \cdot (\rho u \otimes u)) = -\gamma \rho u - \rho \nabla _x V - \rho \nabla _x W \star \rho \\&\quad + \rho \int _{{\mathbb {R}}^d} \psi (x-y) (u(y) - u(x))\,\rho (y)\,\mathrm{d}y, \end{aligned} \end{aligned}$$

in the limit of small inertia $\varepsilon \rightarrow 0$. At the formal level, the equations (1.11) will be replaced by (1.4)–(1.5) as $\varepsilon \rightarrow 0$. The limiting nonlinearly coupled aggregation equations (1.4)–(1.5) have been recently studied in [39, 40]. Several authors have studied particular choices of interactions *V*, *W* and comunication functions $\psi$ for some of the connecting asymptotic limits from the kinetic description (1.10) with/without noise to the hydrodynamic system (1.11) in [8, 11, 42, 57], from the hydrodynamic system (1.11) to the aggregation equation (1.4)–(1.5) in [23, 59, 60], and for the direct limit from the kinetic equation to the aggregation equation (1.4)–(1.5) in [8, 53].

### Purpose, mathematical tools and main novelties

Summarizing the main facts of the mean-field limit and the monokinetic ansatz in Sections 1.1 and 1.2, both the empirical measure $\mu ^N(t)$ associated to the particle system (1.1) and the monokinetic solutions $\rho (x,t) \delta _{u(x,t)}(v)$, with $(\rho ,u)(x,t)$ satisfying the hydrodynamic equations (1.3) in the strong sense, are distributional solutions of the same kinetic equation (1.6). In order to analyse the convergence of the empirical measure $\mu ^N$ to $\rho (x,t) \delta _{u(x,t)}(v)$, the goal is to establish a weak-strong stability estimate where the strong role is played by the distributional solution $\rho (x,t) \delta _{u(x,t)}(v)$ associated to the strong solution of the hydrodynamic system (1.3). Our main goal is then to quantify the following convergence

$$\begin{aligned} \mu ^N_t(x,v) \rightarrow \rho (x,t) \delta _{u(x,t)}(v) \quad \text{ as } \quad N \rightarrow \infty \end{aligned}$$

in the sense of distributions for both the mean-field and the combined mean-field/small inertia limit for well prepared initial data. Our main mathematical tools are the use of a modulated kinetic energy combined with the bounded Lipschitz distance in order to control terms between the discrete particle system and the hydrodynamic quantities. Let us first introduce the modulated kinetic energy as

1.12 $$\begin{aligned} \frac{1}{2} \iint _{{\mathbb {R}}^d \times {\mathbb {R}}^d}f|v - u|^2\,\mathrm{d}x\mathrm{d}v, \end{aligned}$$

where *f* is a solution of kinetic equation (1.6) and *u* is the velocity field as part of the solution of the pressureless Euler equations (1.3). Modulated kinetic energies were used in conjunction with relative potential energy terms for quasineutral limits of Vlasov like equations [5, 6, 62] for instance. We would like to emphasize that the quantity (1.12) gives a sharper estimate compared to the classical modulated macroscopic energy. Indeed, the macro energy of the system (1.3) is given by

$$\begin{aligned} E(U):= \frac{|m|^2}{2 \rho } \quad \text{ with } \quad U:= \begin{pmatrix} \rho \\ m \end{pmatrix}, \quad m = \rho u. \end{aligned}$$

Thus its modulated energy, also often refereed to as relative energy, can be defined as

$$\begin{aligned} E(U_f|U) := E(U_f) - E(U) - D E(U)(U_f - U)\quad \text{ with } \quad U_f := \begin{pmatrix} \rho _f \\ m_f \\ \end{pmatrix}, \quad m_f = \rho _f u _f. \end{aligned}$$

A straightforward computation gives

1.13 $$\begin{aligned} \int _{{\mathbb {R}}^d}E(U_f|U)\,\mathrm{d}x = \frac{1}{2} \int _{{\mathbb {R}}^d}\rho _f |u_f - u|^2\,\mathrm{d}x. \end{aligned}$$

On the other hand, by Hölder inequality, we easily find that

$$\begin{aligned} \rho _f |u_f|^2 \leqq \int _{{\mathbb {R}}^d}|v|^2 f\,\mathrm{d}v. \end{aligned}$$

This yields

$$\begin{aligned} \begin{aligned}&\iint _{{\mathbb {R}}^d \times {\mathbb {R}}^d}f|v-u|^2\,\mathrm{d}x\mathrm{d}v - \int _{{\mathbb {R}}^d}\rho _f |u_f-u|^2\,\mathrm{d}x \\&\quad = \iint _{{\mathbb {R}}^d \times {\mathbb {R}}^d}|v|^2 f\,\mathrm{d}x\mathrm{d}v - \int _{{\mathbb {R}}^d}\rho _f |u_f|^2\,\mathrm{d}x \geqq 0. \end{aligned} \end{aligned}$$

In fact, we can easily show that

1.14 $$\begin{aligned}&\iint _{{\mathbb {R}}^d \times {\mathbb {R}}^d}f|v-u|^2\,\mathrm{d}x\mathrm{d}v = \int _{{\mathbb {R}}^d}\rho _f |u_f-u|^2\,\mathrm{d}x \nonumber \\&\quad + \iint _{{\mathbb {R}}^d \times {\mathbb {R}}^d}f|v - u_f|^2\,\mathrm{d}x\mathrm{d}v. \end{aligned}$$

This shows that the convergence of the modulated kinetic energy (1.12) implies the convergence of the modulated macro energy (1.13). We notice that if *f* is a monokinetic distribution, $f(x,v,t) = \rho _f(x,t) \delta _{u_f(x,t)}(v),$ then the second term on the right hand side of (1.14) becomes zero, and the two modulated energies (1.12) and (1.13) coincide. For notational simplicity, we denote by $\mathcal {Z}^N(t) = \{(x_i(t), v_i(t))\}_{i=1}^N$ the set of trajectories associated to the particle system (1.1). Then let us define the first important quantity that will allow us to quantify the distance between particles (1.1) and hydrodynamics (1.3), it is just the discrete version of the modulated kinetic energy (1.12) defined as

1.15 $$\begin{aligned}&\mathcal {E}^N (\mathcal {Z}^N(t) | U(t)) := \frac{1}{2} \iint _{{\mathbb {R}}^d \times {\mathbb {R}}^d}|u-v|^2\,\mu ^N_t(\mathrm{d}x\mathrm{d}v) \nonumber \\&\quad = \frac{1}{2N} \sum _{i=1}^N |u(x_i(t),t) - v_i(t)|^2. \end{aligned}$$

The second quantity that will allow us our quantification goal combined with the discrete modulated energy (1.15) is a classical distance between probability measures, the bounded Lipschitz distance, used already by the pioneers in kinetic theory [4, 64, 67] in the early works for the mean-field limit. Notice that the pressureless Euler system (1.3) includes the nonlocal position and velocity interaction and alignment forces. Furthermore, its relative energy/entropy has no strict convexity in terms of density variable due to the lack of pressure term. In order to overcome these difficulties, ideas of combining the modulated macro energy and the first or second order Wasserstein distance have been recently proposed in [8, 11, 32] quantifying the hydrodynamic limit from kinetic equation to the pressureless Euler type system. More recently, in [24], a general theory providing some relation between a modulated macro energy-type function and *p*-Wasserstein distance is also developed. In particular, in [24, Proposition 3.1], it is discussed that the *p*-Wasserstein distance with $p\in [1,2]$ can be controlled by the modulated macro energy functional.

In the present work, we will employ the bounded Lipschitz distance to provide stability estimates between the empirical particle density $\rho ^N$ defined as

$$\begin{aligned} \rho ^N_t(x) := \int _{{\mathbb {R}}^d} \mu ^N_t\,(\mathrm{d}v) = \frac{1}{N} \sum _{j=1}^N \delta _{x_j(t)}(x) \end{aligned}$$

with $\mu ^N_t$ be the empirical measure associated to the particle system (1.1), and the hydrodynamic particle density $\rho$ solution to (1.3). More precisely, let ${\mathcal {M}}({\mathbb {R}}^d)$ be the space of signed Radon measures on ${\mathbb {R}}^d$, which can be considered as nonnegative bounded linear functionals on ${\mathcal {C}}_0({\mathbb {R}}^d)$. Let $\mu , \nu \in {\mathcal {M}}({\mathbb {R}}^d)$ be two Radon measures. Then the bounded Lipschitz distance, which is denoted by $d_{BL}: {\mathcal {M}}({\mathbb {R}}^d) \times {\mathcal {M}}({\mathbb {R}}^d) \rightarrow {\mathbb {R}}_+$, between $\mu$ and $\nu$ is defined by

$$\begin{aligned} d_{BL}(\mu ,\nu ) := \sup _{\phi \in \Omega } \left| \int _{{\mathbb {R}}^d}\phi (x)( \mu (\mathrm{d}x) - \nu (\mathrm{d}x))\right| , \end{aligned}$$

where the admissible set $\Omega$ of test functions are given by

$$\begin{aligned} \Omega := \left\{ \phi : {\mathbb {R}}^d \rightarrow {\mathbb {R}}: \Vert \phi \Vert _{L^\infty } \leqq 1, \ Lip(\phi ) := \sup _{x \ne y} \frac{|\phi (x) - \phi (y)|}{|x-y|} \leqq 1 \right\} . \end{aligned}$$

We also denote by $Lip({\mathbb {R}}^d)$ the set of Lipschitz functions on ${\mathbb {R}}^d$. In Proposition 2.2 below, we provide a relation between the bounded Lispchitz distance and the discrete version of the modulated kinetic energy (1.15). This key observation allows us to overcome the difficulties mentioned above.

### Main results and Plan of the paper

We will first assume that the particle system (1.1), the pressureless Euler-type equations (1.3), and the aggregation equations (1.4)–(1.5) have existence of smooth enough solutions up to a fixed time $T>0$. We postpone further discussion at the end of this subsection, although we make precise now the assumptions needed on these solutions for our main results.

Our first main result shows the rigorous passage from Newton’s equation (1.1) to pressureless Euler equations (1.3) via the mean-field limit as $N \rightarrow \infty$.

#### Theorem 1.1

Let $T > 0$, $\mathcal {Z}^N(t) = \{(x_i(t), v_i(t))\}_{i=1}^N$ be a solution to the particle system (1.1), and let $(\rho , u)$ be the unique classical solution of the pressureless Euler system with nonlocal interaction forces (1.3) satisfying $\rho >0$ on ${\mathbb {R}}^d \times [0,T)$, $\rho \in {\mathcal {C}}([0,T];{\mathcal {P}}({\mathbb {R}}^d))$ and $u \in L^\infty (0,T;\mathcal {W}^{1,\infty }({\mathbb {R}}^d))$ up to time $T>0$ with initial data $(\rho _0, u_0)$. Suppose that the interaction potential *W* and the communication weight function $\psi$ satisfy $\nabla _x W \in \mathcal {W}^{1,\infty }({\mathbb {R}}^d)$ and $\psi \in \mathcal {W}^{1,\infty }({\mathbb {R}}^d)$, respectively. If the initial data for (1.1) and (1.3) are chosen such that

$$\begin{aligned} \iint _{{\mathbb {R}}^d \times {\mathbb {R}}^d}|v - u_0(x) |^2\mu ^N_0(\mathrm{d}x\mathrm{d}v) + d^2_{BL}(\rho ^N_0, \rho _0) \rightarrow 0 \quad \text{ as } \quad N \rightarrow \infty , \end{aligned}$$

then we have

$$\begin{aligned} \begin{aligned} \int _{{\mathbb {R}}^d}v \,\mu ^N(\mathrm{d}v) = \frac{1}{N}\sum _{i=1}^N v_i \,\delta _{x_i}&\rightharpoonup \rho u \quad \text{ weakly } \text{ in } L^\infty (0,T;{\mathcal {M}}({\mathbb {R}}^d)), \\ \int _{{\mathbb {R}}^d}(v \otimes v)\, \mu ^N(\mathrm{d}v) = \frac{1}{N}\sum _{i=1}^N (v_i \otimes v_i)\,\delta _{x_i}&\rightharpoonup \rho u \otimes u \quad \text{ weakly } \text{ in } L^\infty (0,T;{\mathcal {M}}({\mathbb {R}}^d)), \quad \text{ and }\\ \mu ^N&\rightharpoonup \rho \delta _{u} \quad \text{ weakly } \text{ in } L^\infty (0,T;{\mathcal {M}}({\mathbb {R}}^d \times {\mathbb {R}}^d)) \end{aligned} \end{aligned}$$

as $N \rightarrow \infty$. In fact, we have the following quantitative bound estimate:

$$\begin{aligned} \begin{aligned}&\iint _{{\mathbb {R}}^d \times {\mathbb {R}}^d}|v - u(x,t) |^2 \mu ^N_t(\mathrm{d}x\mathrm{d}v) \,+ d^2_{BL}(\rho ^N_t(\cdot ), \rho (\cdot ,t)) \\&\quad \leqq C\left( \iint _{{\mathbb {R}}^d \times {\mathbb {R}}^d}|v - u_0(x) |^2\mu ^N_0(\mathrm{d}x\mathrm{d}v) + d^2_{BL}(\rho ^N_0, \rho _0)\right) , \end{aligned} \end{aligned}$$

where $C>0$ only depends on $\Vert u\Vert _{L^\infty (0,T;\mathcal {W}^{1,\infty })}$, $\Vert \psi \Vert _{\mathcal {W}^{1,\infty }}$, $\Vert \nabla _x W\Vert _{\mathcal {W}^{1,\infty }}$, and *T*.

The main novelty of this first result resides in how to control the alignment terms via the modulated energy combined with the bounded Lipschitz distance.

#### Remark 1.1

(Singular repulsive interaction) The previous result also applies to singular repulsive interaction potentials. In particular, it holds for the Coulomb interaction potential on ${\mathbb {R}}^d$ given by

$$\begin{aligned} {\mathcal {N}}(x) = \left\{ \begin{array}{ll} \displaystyle -\frac{|x|}{2} &{} \text {for } d=1,\\ \displaystyle -\frac{1}{2\pi } \log |x| &{} \text {for } d=2,\\ \displaystyle \frac{1}{d(d-2)\alpha _d}\frac{1}{|x|^{d-2}} &{} \text {for } d \geqq 3, \end{array} \right. \end{aligned}$$

and for Riesz potentials in a sense to be specified in Section 2.3. Here $\alpha _d$ denotes the volume of the unit ball in ${\mathbb {R}}^d$. In order to deal with the singularity on the interaction potential, the diagonal term should be eliminated in the modulated energy functional. This has been recently solved in the recent breakthrough result in [66] by introducing a different relative potential energy avoiding the diagonal terms. The details for singular interaction potentials cases are postponed to Section 2.3, see Theorem 2.1.

Section 2 is devoted to the proof of Theorem 1.1 and the generalization to singular repulsive potentials using [66] in its last subsection.

Our second main result is devoted to the asymptotic analysis for the particle system (1.1) under the small inertia regime: $\varepsilon _N \rightarrow 0$ as $N \rightarrow \infty$. By Theorem 1.1, we expect that for sufficiently large $N\gg 1$, the system (1.1) in the mean-field/small inertia limit can be well approximated by

$$\begin{aligned} \begin{aligned}&\partial _t {\bar{\rho }} + \nabla _x \cdot ({\bar{\rho }} {\bar{u}}) = 0,\\&\qquad \varepsilon _N\partial _t ({\bar{\rho }} {\bar{u}}) + \varepsilon _N\nabla _x \cdot ({\bar{\rho }} {\bar{u}} \otimes {\bar{u}}) \\&\quad = -\gamma {\bar{\rho }} {\bar{u}} - {\bar{\rho }} \nabla _x V - {\bar{\rho }} \nabla _x W \star {\bar{\rho }} + {\bar{\rho }} \int _{{\mathbb {R}}^d} \psi (x-y) ({\bar{u}}(y) - {\bar{u}}(x))\,{\bar{\rho }}(y)\,\mathrm{d}y. \end{aligned} \end{aligned}$$

At the formal level, since $\varepsilon _N \rightarrow 0$ as $N \rightarrow \infty$, it follows from the momentum equations in the above system that the hydrodynamic system (1.3) should be replaced by (1.4)–(1.5) as $N \rightarrow \infty$. In order to apply our strategy above, we rewrite the equations (1.4)–(1.5) as

1.16 $$\begin{aligned} \begin{aligned}&\partial _t {\bar{\rho }} + \nabla _x \cdot ({\bar{\rho }} {\bar{u}}) = 0,\\&\quad \varepsilon _N\partial _t ({\bar{\rho }} {\bar{u}}) + \varepsilon _N\nabla _x \cdot ({\bar{\rho }} {\bar{u}} \otimes {\bar{u}}) = -\gamma {\bar{\rho }} {\bar{u}} - {\bar{\rho }} \nabla _x V - {\bar{\rho }} \nabla _x W \star {\bar{\rho }} \\&\qquad + {\bar{\rho }} \int _{{\mathbb {R}}^d} \psi (x-y) ({\bar{u}}(y) - {\bar{u}}(x))\,{\bar{\rho }}(y)\,\mathrm{d}y + \varepsilon _N {\bar{\rho }} {\bar{e}}, \end{aligned} \end{aligned}$$

where $${\bar{e}} := \partial _t {\bar{u}} + {\bar{u}} \cdot \nabla _x {\bar{u}}$$.

We can now state our second main result related to a weak-strong stability estimate in the combined mean-field/small inertia limit.

#### Theorem 1.2

Let $T>0$ and $d \geqq 1$. Let $\mathcal {Z}^N(t) = \{(x_i(t), v_i(t))\}_{i=1}^N$ be a solution to the particle system (1.1), and let $({\bar{\rho }}, {\bar{u}})$ be the unique classical solution of the aggregation-type equation (1.4)–(1.5) satisfying ${\bar{\rho }} \in {\mathcal {C}}([0,T];{\mathcal {P}}({\mathbb {R}}^d))$ and ${\bar{\rho }} > 0$ on ${\mathbb {R}}^d \times [0,T)$, ${\bar{u}} \in L^\infty (0,T; \mathcal {W}^{1,\infty }({\mathbb {R}}^d))$ and $\partial _t {\bar{u}} \in L^\infty ({\mathbb {R}}^d \times (0,T))$ up to time $T>0$ with the initial data ${\bar{\rho }}_0$. Suppose that the interaction potential *W* and the communication weight function $\psi$ satisfy $\nabla _x W \in \mathcal {W}^{1,\infty }({\mathbb {R}}^d)$ and $\psi \in \mathcal {W}^{1,\infty }({\mathbb {R}}^d)$, respectively, and the strength of damping $\gamma >0$ is large enough. If the initial data for (1.1) and (1.4) are chosen such that

$$\begin{aligned} \iint _{{\mathbb {R}}^d \times {\mathbb {R}}^d}|v - {\bar{u}}_0(x) |^2\mu ^N_0(\mathrm{d}x\mathrm{d}v) + d_{BL}(\rho ^N_0,{\bar{\rho }}_0) \rightarrow 0 \quad \text{ as } \quad N \rightarrow \infty , \end{aligned}$$

then we have

1.17 $$\begin{aligned} \int _{{\mathbb {R}}^d}v \,\mu ^N(\mathrm{d}v) = \frac{1}{N}\sum _{i=1}^N v_i \,\delta _{x_i} \rightharpoonup {\bar{\rho }} {\bar{u}} \quad \text{ weakly } \text{ in } L^1(0,T;{\mathcal {M}}({\mathbb {R}}^d)) \end{aligned}$$

and

1.18 $$\begin{aligned} \mu ^N \rightharpoonup {\bar{\rho }}\delta _{{\bar{u}}} \quad \text{ weakly } \text{ in } L^1(0,T;{\mathcal {M}}({\mathbb {R}}^d \times {\mathbb {R}}^d)) \end{aligned}$$

as $N \rightarrow \infty$ (and thus $\varepsilon _N \rightarrow 0$). In fact, we have the following quantitative bound estimate:

$$\begin{aligned} \begin{aligned}&d_{BL}^2(\rho ^N_t(\cdot ),{\bar{\rho }}(\cdot ,t)) +\int _0^t \iint _{{\mathbb {R}}^d \times {\mathbb {R}}^d}|v - {\bar{u}}(x,s) |^2\mu ^N_s(\mathrm{d}x\mathrm{d}v) \,\mathrm{d}s \\&\quad \leqq C\varepsilon _N\iint _{{\mathbb {R}}^d \times {\mathbb {R}}^d}|v - {\bar{u}}_0(x) |^2\mu ^N_0(\mathrm{d}x\mathrm{d}v) +Cd_{BL}^2(\rho ^N_0,{\bar{\rho }}_0) + C\varepsilon _N^2 \end{aligned} \end{aligned}$$

and

$$\begin{aligned} \begin{aligned}&\frac{1}{\varepsilon _N}d^2_{BL}(\rho ^N_t(\cdot ),{\bar{\rho }}(\cdot ,t)) + \iint _{{\mathbb {R}}^d \times {\mathbb {R}}^d}|v - {\bar{u}}(x,t) |^2\mu ^N_t(\mathrm{d}x\mathrm{d}v) \\&\quad \leqq C(1 + \varepsilon _N)\iint _{{\mathbb {R}}^d \times {\mathbb {R}}^d}|v - {\bar{u}}_0(x) |^2\mu ^N_0(\mathrm{d}x\mathrm{d}v) + \frac{C}{\varepsilon _N}d_{BL}^2(\rho ^N_0,{\bar{\rho }}_0) + C\varepsilon _N \end{aligned} \end{aligned}$$

for all $t \in [0,T]$, where $C>0$ is independent of both $\varepsilon _N$ and *N* but depending on $\Vert {\bar{u}}\Vert _{L^\infty (0,T;\mathcal {W}^{1,\infty })}$, $\Vert \partial _t {\bar{u}}\Vert _{L^\infty }$, $\Vert \nabla _x W\Vert _{\mathcal {W}^{1,\infty }}$, $\Vert \psi \Vert _{\mathcal {W}^{1,\infty }}$, and $\gamma$.

#### Remark 1.2

Theorem 1.2 implies that if the initial data satisfies

$$\begin{aligned} \iint _{{\mathbb {R}}^d \times {\mathbb {R}}^d}|v - {\bar{u}}_0(x) |^2\mu ^N_0(\mathrm{d}x\mathrm{d}v) + d_{BL}(\rho ^N_0,{\bar{\rho }}_0) \leqq C_0 \,\varepsilon _N \end{aligned}$$

for some $C_0>0$ which is independent of both $\varepsilon _N$ and *N*, then we have

$$\begin{aligned} d_{BL}^2(\rho ^N_t(\cdot ),{\bar{\rho }}(\cdot ,t)) + \int _0^t \iint _{{\mathbb {R}}^d \times {\mathbb {R}}^d}|v - {\bar{u}}(x,s) |^2\mu ^N_s(\mathrm{d}x\mathrm{d}v) \,ds \leqq C\varepsilon _N^2 \end{aligned}$$

and

$$\begin{aligned} \iint _{{\mathbb {R}}^d \times {\mathbb {R}}^d}|v - {\bar{u}}(x,t) |^2\mu ^N_t(\mathrm{d}x\mathrm{d}v) \leqq C\varepsilon _N \end{aligned}$$

for all $t \in [0,T]$, where $C>0$ is independent of both $\varepsilon _N$ and *N*. This further yields that the convergences (1.17) and (1.18) hold in weakly in $L^\infty (0,T;{\mathcal {M}}({\mathbb {R}}^d))$ and $L^\infty (0,T;{\mathcal {M}}({\mathbb {R}}^d \times {\mathbb {R}}^d))$, respectively.

#### Remark 1.3

If $V\equiv 0$ and $\gamma >0$ is sufficiently large, then we can check that $\Vert {\bar{u}}\Vert _{L^\infty (0,T;\mathcal {W}^{1,\infty })}$ and $\Vert \partial _t {\bar{u}}\Vert _{L^\infty }$ can be bounded from above by some constant, which depends only on $\Vert \nabla _x W\Vert _{\mathcal {W}^{1,\infty }}$, $\Vert \psi \Vert _{\mathcal {W}^{1,\infty }}$, $\Vert {\bar{\rho }}\Vert _{L^\infty (0,T;L^1)}$, and $\gamma$. We refer to [24] for details. For general confinement potentials, we can also deal with general strong solutions for compactly supported initial data since their support remains compact for all times. We refer to [1, 15] for particular instances of these results.

#### Remark 1.4

One may follow a similar argument as in [40, Theorem 2.4] to have the existence and uniqueness of classical solutions $({\bar{\rho }}, {\bar{u}})$ to the equations (1.4)–(1.5) satisfying the regularity properties and assumptions of Theorem 1.2. For the Coulomb or Riesz interaction, an idea of proof proposed in [28] would be employed to establish the local-in-time existence and uniqueness of classical solutions to the equations (1.4)–(1.5) without the confinement potential.

Section 3 is devoted to the proof of Theorem 1.2 and the generalizations to singular repulsive potentials. Finally, we complement these results by showing the existence of solutions to the particle system (1.1) in Appendix A, and the existence and uniqueness of classical solutions stated in Theorem 1.1 for the hydrodynamic system (1.3) in Section 4.

## Mean-Field Limit: From Newton to Pressureless Euler

In this section, we provide the details of the proof for Theorem 1.1. As mentioned before, one of our main mathematical tools is the discrete version of the modulated kinetic energy $\mathcal {E}^N (\mathcal {Z}^N(t) | U(t))$ defined in (1.15).

### Modulated kinetic energy estimate

In this part, our main purpose is to give the quantitative bound estimate of the discrete modulated kinetic energy $\mathcal {E}^N (\mathcal {Z}^N(t) | U(t))$.

#### Proposition 2.1

Let $T > 0$, $\mathcal {Z}^N(t) = \{(x_i(t), v_i(t))\}_{i=1}^N$ be a solution to the particle system (1.1), and let $(\rho , u)$ be the unique classical solution of the pressureless Euler system with nonlocal interaction forces (1.3) under the assumptions of Theorem 1.1 up to time $T>0$. Suppose that the interaction potential *W* and the communication weight function $\psi$ satisfy $\nabla _x W \in \mathcal {W}^{1,\infty }({\mathbb {R}}^d)$ and $\psi \in \mathcal {W}^{1,\infty }({\mathbb {R}}^d)$, respectively. Then we have

2.1 $$\begin{aligned} \begin{aligned}&\frac{\mathrm{d}}{\mathrm{d}t} \mathcal {E}^N (\mathcal {Z}^N(t) | U(t))+ 2\gamma \mathcal {E}^N (\mathcal {Z}^N(t) | U(t)) + \frac{1}{N^2} \sum _{i,j=1}^N \psi (x_i - x_j)|v_i - u(x_i)|^2 \\&\quad \leqq C\mathcal {E}^N (\mathcal {Z}^N(t) | U(t)) + C d_{BL}^2(\rho ^N_t(\cdot ),\rho (\cdot ,t)), \end{aligned} \end{aligned}$$

where $C>0$ is independent of *N* and $\gamma$.

#### Proof

By the notion of our classical solution, we obtain from the momentum equation in (1.3) that

$$\begin{aligned} \partial _t (u(x_i(t),t))= & {} v_i(t) \cdot \nabla _x u(x_i(t),t) + (\partial _t u)(x_i(t),t)\\= & {} (v_i(t) - u(x_i(t),t)) \cdot \nabla _x u(x_i(t),t) - \gamma u(x_i(t)) \\&- \nabla _x V(x_i(t)) - (\nabla _x W \star \rho )(x_i) \\&+ \int _{{\mathbb {R}}^d} \psi (x_i(t) - y) (u(y,t) - u(x_i(t),t) )\rho (y,t)\,\mathrm{d}y. \end{aligned}$$

Then using this and (1.1), we estimate the discrete modulated kinetic energy functional as

2.2 $$\begin{aligned}&\frac{\mathrm{d}}{\mathrm{d}t} \mathcal {E}^N (\mathcal {Z}^N(t) | U(t)) = \, \frac{1}{N} \sum _{i=1}^N (u(x_i(t),t) - v_i(t)) \nonumber \\&\qquad \cdot \left( \partial _t u(x_i(t),t) + v_i(t) \cdot \nabla _x u(x_i(t),t) - \dot{v}_i(t)\right) \nonumber \\&\quad =\,\frac{1}{N} \sum _{i=1}^N (u(x_i(t),t) - v_i(t)) \cdot ((v_i(t) - u(x_i(t),t))\cdot \nabla _x)u(x_i(t),t) \nonumber \\&\qquad - \frac{\gamma }{N} \sum _{i=1}^N |u(x_i(t),t) - v_i(t)|^2 \nonumber \\&\qquad - \frac{1}{N} \sum _{i=1}^N (u(x_i(t),t) - v_i(t)) \cdot \left( (\nabla _x W \star \rho )(x_i) - (\nabla _x W \star \rho ^N)(x_i)\right) \nonumber \\&\qquad + \frac{1}{N} \sum _{i=1}^N (u(x_i(t),t) - v_i(t)) \cdot F(x_i(t), v_i(t))\nonumber \\&\quad =: \,\sum _{i=1}^4 I_i, \end{aligned}$$

where

$$\begin{aligned} \begin{aligned}&F(x_i(t), v_i(t)):= \int _{{\mathbb {R}}^d} \psi (x_i(t) - y) (u(y,t) - u(x_i(t),t) )\rho (y,t)\,\mathrm{d}y \\&\quad - \frac{1}{N} \sum _{j=1}^N \psi (x_i(t) - x_j(t))(v_j(t) - v_i(t)). \end{aligned} \end{aligned}$$

Here $I_1$ can be easily estimated as

$$\begin{aligned} \begin{aligned} I_1&= \frac{1}{N} \sum _{i=1}^N \nabla _x u(x_i(t),t) : (u(x_i(t),t) - v_i(t)) \otimes (v_i(t) - u(x_i(t),t))\\&\leqq \Vert \nabla _x u(\cdot ,t)\Vert _{L^\infty }\frac{1}{N} \sum _{i=1}^N |u(x_i(t),t) - v_i(t)|^2 \\&= 2 \Vert \nabla _x u(\cdot ,t)\Vert _{L^\infty }\mathcal {E}^N (\mathcal {Z}^N(t) | U(t)). \end{aligned} \end{aligned}$$

By definition, we obtain $I_2 = -2\gamma \mathcal {E}^N (\mathcal {Z}^N(t) | U(t)).$ We next estimate $I_3$ as

$$\begin{aligned} \begin{aligned} I_3&= - \frac{1}{N} \sum _{i=1}^N (u(x_i(t),t) {-} v_i(t)) \cdot \left( (\nabla _x W \star \rho )(x_i(t),t) {-} (\nabla _x W \star \rho ^N)(x_i(t),t) \right) \\&= \frac{1}{N} \sum _{i=1}^N (v_i(t) - u(x_i(t),t)) \cdot (\nabla _x W \star (\rho - \rho ^N))(x_i(t),t). \end{aligned} \end{aligned}$$

On the other hand, the fact $\nabla _x W \in \mathcal {W}^{1,\infty }$ gives

$$\begin{aligned} \Vert (\nabla _x W \star (\rho - \rho ^N))(\cdot ,t)\Vert _{L^\infty } \leqq \Vert \nabla _x W\Vert _{\mathcal {W}^{1,\infty }}d_{BL}(\rho ^N,\rho ), \end{aligned}$$

and subsequently this asserts

$$\begin{aligned} \begin{aligned} I_3&\leqq \Vert \nabla _x W\Vert _{\mathcal {W}^{1,\infty }}d_{BL}(\rho ^N,\rho )\left( \frac{1}{N} \sum _{i=1}^N |v_i(t) - u(x_i(t),t)| \right) \\&\leqq \Vert \nabla _x W\Vert _{\mathcal {W}^{1,\infty }}d_{BL}(\rho ^N,\rho )\left( \frac{1}{N} \sum _{i=1}^N |v_i(t) - u(x_i(t),t)|^2 \right) ^{1/2} \\&=\Vert \nabla _x W\Vert _{\mathcal {W}^{1,\infty }}d_{BL}(\rho ^N,\rho ) \sqrt{\mathcal {E}^N (\mathcal {Z}^N(t) | U(t))}. \end{aligned} \end{aligned}$$

For the estimate of $I_4$, we note that

$$\begin{aligned} \begin{aligned}&\frac{1}{N} \sum _{j=1}^N \psi (x_i(t) - x_j(t))(v_j(t) - v_i(t))\\&\quad = \frac{1}{N} \sum _{j=1}^N \psi (x_i(t) - x_j(t))(v_j(t) - u(x_j(t),t)) \\&\qquad + \frac{1}{N} \sum _{j=1}^N \psi (x_i(t) - x_j(t))(u(x_j(t),t) - v_i(t))\\&\quad =: J_1 + J_2. \end{aligned} \end{aligned}$$

Then we rewrite $J_2$ as

$$\begin{aligned} J_2 = \int _{{\mathbb {R}}^d} \psi (x_i(t) - y) (u(y,t) - v_i(t))\rho ^N(y,t)\,\mathrm{d}y. \end{aligned}$$

This yields

$$\begin{aligned} \begin{aligned} I_4&= \frac{1}{N} \sum _{i=1}^N (u(x_i) - v_i) \cdot \frac{1}{N} \sum _{j=1}^N \psi (x_i - x_j)(u(x_j) - v_j)\\&\quad + \frac{1}{N} \sum _{i=1}^N (u(x_i) - v_i) \\&\quad \cdot \left( \int _{{\mathbb {R}}^d} \psi (x_i - y) (u(y) - u(x_i) )\rho (y)\,\mathrm{d}y - \int _{{\mathbb {R}}^d} \psi (x_i - y) (u(y) - v_i)\rho ^N(y)\,\mathrm{d}y \right) \\&=: I_4^1 + I_4^2. \end{aligned} \end{aligned}$$

Here we can easily estimate $I_4^1$ as

$$\begin{aligned} I_4^1\leqq & {} \Vert \psi \Vert _{L^\infty }\left( \frac{1}{N} \sum _{i=1}^N (u(x_i) - v_i) \right) ^2 \leqq \Vert \psi \Vert _{L^\infty }\frac{1}{N} \sum _{i=1}^N |u(x_i) - v_i|^2 \\= & {} 2 \Vert \psi \Vert _{L^\infty }\mathcal {E}^N (\mathcal {Z}^N(t) | U(t)). \end{aligned}$$

Note that

$$\begin{aligned} \begin{aligned}&\frac{1}{N}\sum _{i=1}^N \int _{{\mathbb {R}}^d} \psi (x_i - y)(v_i - u(x_i)) (\rho ^N(y) - \rho (y)) \cdot (u(y) - u(x_i))\,\mathrm{d}y\\&\quad = \frac{1}{N}\sum _{i=1}^N \int _{{\mathbb {R}}^d} \psi (x_i - y)(v_i - u(x_i)) \rho ^N(y) \cdot (u(y) - u(x_i))\,\mathrm{d}y\\&\qquad + I_4^2 - \frac{1}{N}\sum _{i=1}^N \int _{{\mathbb {R}}^d} \psi (x_i - y)(v_i - u(x_i)) \rho ^N(y) \cdot (u(y) - v_i)\,\mathrm{d}y\\&\quad = I_4^2 + \frac{1}{N^2} \sum _{i,j=1}^N \psi (x_i - x_j)|v_i - u(x_i)|^2, \end{aligned} \end{aligned}$$

that is,

$$\begin{aligned} \begin{aligned} I_4^2&= \frac{1}{N}\sum _{i=1}^N \int _{{\mathbb {R}}^d} \psi (x_i - y)(v_i - u(x_i)) (\rho ^N(y) - \rho (y)) \cdot (u(y) - u(x_i))\,\mathrm{d}y\\&\quad - \frac{1}{N^2} \sum _{i,j=1}^N \psi (x_i - x_j)|v_i - u(x_i)|^2. \end{aligned} \end{aligned}$$

On the other hand, we can estimate

$$\begin{aligned} \begin{aligned}&\frac{1}{N}\sum _{i=1}^N \int _{{\mathbb {R}}^d} \psi (x_i - y)(v_i - u(x_i)) (\rho ^N(y) - \rho (y)) \cdot (u(y) - u(x_i))\,\mathrm{d}y\\&\quad = \frac{1}{N}\sum _{i=1}^N (v_i - u(x_i)) \cdot \int _{{\mathbb {R}}^d} \psi (x_i - y)u(y) (\rho ^N(y) - \rho (y)) \,\mathrm{d}y\\&\qquad - \frac{1}{N}\sum _{i=1}^N(v_i - u(x_i)) \cdot u(x_i) \int _{{\mathbb {R}}^d} \psi (x_i - y) (\rho ^N(y) - \rho (y)) \,\mathrm{d}y\\&\quad =: K_1 + K_2, \end{aligned} \end{aligned}$$

where

$$\begin{aligned} \begin{aligned} \left| K_1\right|&\leqq \frac{1}{N}\sum _{i=1}^N |v_i - u(x_i)| \left| \int _{{\mathbb {R}}^d} \psi (x_i - y)u(y) (\rho ^N(y) - \rho (y)) \,\mathrm{d}y\right| \\&\leqq \Vert \psi u\Vert _{\mathcal {W}^{1,\infty }}\frac{1}{N}\sum _{i=1}^N |v_i - u(x_i)| \,d_{BL}(\rho ^N,\rho )\\&\leqq \Vert \psi u\Vert _{\mathcal {W}^{1,\infty }}\left( \frac{1}{N}\sum _{i=1}^N |v_i - u(x_i)|^2\right) ^{1/2}d_{BL}(\rho ^N,\rho )\\&\leqq \Vert \psi u\Vert _{\mathcal {W}^{1,\infty }}\sqrt{2} \sqrt{\mathcal {E}^N (\mathcal {Z}^N(t) | U(t))} \,d_{BL}(\rho ^N,\rho ). \end{aligned} \end{aligned}$$

Similarly, we also find that

$$\begin{aligned} \begin{aligned} \left| K_2\right|&\leqq \frac{1}{N}\sum _{i=1}^N |v_i - u(x_i)| |u(x_i)|\left| \int _{{\mathbb {R}}^d} \psi (x_i - y) (\rho ^N(y) - \rho (y)) \,\mathrm{d}y\right| \\&\leqq \Vert u\Vert _{L^\infty }\Vert \psi \Vert _{\mathcal {W}^{1,\infty }}\sqrt{2} \sqrt{\mathcal {E}^N (\mathcal {Z}^N(t) | U(t))} \,d_{BL}(\rho ^N,\rho ). \end{aligned} \end{aligned}$$

Combining all of the above estimates, we have

$$\begin{aligned} \begin{aligned}&\frac{\mathrm{d}}{\mathrm{d}t} \mathcal {E}^N (\mathcal {Z}^N(t) | U(t))+ 2\gamma \mathcal {E}^N (\mathcal {Z}^N(t) | U(t)) + \frac{1}{N^2} \sum _{i,j=1}^N \psi (x_i - x_j)|v_i - u(x_i)|^2 \\&\quad \leqq 2\left( \Vert \nabla _x u(\cdot ,t)\Vert _{L^\infty } + \Vert \psi \Vert _{L^\infty }\right) \mathcal {E}^N (\mathcal {Z}^N(t) | U(t)) \\&\qquad + \sqrt{2}\left( \Vert \psi u\Vert _{\mathcal {W}^{1,\infty }}+ \Vert u(\cdot ,t)\Vert _{L^\infty }\Vert \psi \Vert _{\mathcal {W}^{1,\infty }} + \Vert \nabla _x W\Vert _{\mathcal {W}^{1,\infty }}\right) \\&\qquad \sqrt{\mathcal {E}^N (\mathcal {Z}^N(t) | U(t))} \,d_{BL}(\rho ^N_t(\cdot ),\rho (\cdot ,t)). \end{aligned} \end{aligned}$$

This completes the proof. $\quad \square$

#### Remark 2.1

We assumed that the communication weight $\psi$ is nonnegative, which takes into account the velocity alignment forces, however a similar bound estimate for the discrete kinetic energy $\mathcal {E}^N$ to that in Proposition 2.1 can be obtained. Indeed, if $\psi$ can be negative, but bounded, then the third term on the left hand side of (2.1) can be estimated as

$$\begin{aligned} \left| \frac{1}{N^2} \sum _{i,j=1}^N \psi (x_i - x_j)|v_i - u(x_i)|^2\right| \leqq 2\Vert \psi \Vert _{L^\infty } \mathcal {E}^N (\mathcal {Z}^N | U). \end{aligned}$$

This yields

$$\begin{aligned} \frac{\mathrm{d}}{\mathrm{d}t} \mathcal {E}^N (\mathcal {Z}^N(t) | U(t))+ 2\gamma \mathcal {E}^N (\mathcal {Z}^N(t) | U(t))\leqq C\mathcal {E}^N (\mathcal {Z}^N(t) | U(t)) + C d_{BL}^2(\rho ^N_t(\cdot ),\rho (\cdot ,t)), \end{aligned}$$

where $C>0$ is independent of *N* and $\gamma$.

In order to close the estimate in Proposition 2.1, we need to estimate the bounded Lipschitz distance between $\rho ^N$ and $\rho$. In the proposition below, we provide the relation between the bounded Lipschitz distance and the discrete modulated kinetic energy.

#### Proposition 2.2

Let $\rho ^N$ and $\rho$ be defined as above. Then we have

$$\begin{aligned} d_{BL}^2(\rho ^N(\cdot ,t),\rho (\cdot ,t)) \leqq Cd_{BL}^2(\rho ^N_0,\rho _0) + C\int _0^t \mathcal {E}^N (\mathcal {Z}^N(s) | U(s))\,\mathrm{d}s, \end{aligned}$$

where $C > 0$ depends only on $\Vert u\Vert _{L^\infty (0,T;Lip)}$ and *T*.

#### Proof

Consider a forward characteristics $\eta =\eta (x,t)$ for the system (1.3) satisfying the following ODEs:

2.3 $$\begin{aligned} \frac{\mathrm{d}\eta (x,t)}{\mathrm{d}t} = u(\eta (x,t),t) \end{aligned}$$

subject to the initial data: $\eta (x,0) = x \in {\mathbb {R}}^d.$ The characteristic $\eta$ is well-defined because of the Lipschitz continuous regularity of *u*. Note that along the characteristic, the solution $\rho$ can be written in the mild form

$$\begin{aligned} \rho (\eta (x,t),t) = \rho _0(x) \exp \left( -\int _0^t (\nabla _x \cdot u)(\eta (x,s),s)\,\mathrm{d}s\right) , \end{aligned}$$

and thus we get

$$\begin{aligned} \rho _0(x) = \rho (\eta (x,t),t)\exp \left( \int _0^t (\nabla _x \cdot u)(\eta (x,s),s)\,\mathrm{d}s\right) = \rho (\eta (x,t),t) det\left( (\nabla _x \eta )(x,t) \right) . \end{aligned}$$

This together with using the change of variables yields

2.4 $$\begin{aligned} \int _{{\mathbb {R}}^d}\phi (\eta (x,t)) \rho _0(x)\,\mathrm{d}x= & {} \int _{{\mathbb {R}}^d}\phi (\eta (x,t)) \rho (\eta (x,t),t) det\left( (\nabla _x \eta )(x,t) \right) \,\mathrm{d}x \nonumber \\= & {} \int _{{\mathbb {R}}^d}\phi (x) \rho (x,t)\,\mathrm{d}x \end{aligned}$$

for $\phi \in \mathcal {W}^{1,\infty }({\mathbb {R}}^d)$. Moreover, we find from (2.3) that

2.5 $$\begin{aligned} \begin{aligned} \left| \eta (x,t) - \eta (y,t)\right|&= \left| x- y + \int _0^t \left( u(\eta (x,s),s) - u(\eta (y,s),s)\right) \,\mathrm{d}s \right| \\&\leqq |x-y| + \Vert u\Vert _{Lip} \int _0^t |\eta (x,s) - \eta (y,s)|\,\mathrm{d}s, \end{aligned} \end{aligned}$$

and applying Grönwall’s lemma to the above gives

$$\begin{aligned} \left| \eta (x,t) - \eta (y,t)\right| \leqq C|x - y|, \end{aligned}$$

where $C>0$ depends only on $\Vert u\Vert _{L^\infty (0,T;Lip)}$ and *T*, that is, $\eta$ is Lipschitz continuous in ${\mathbb {R}}^d$. We also get

$$\begin{aligned} |x_i(t) - \eta (x,t)| \leqq |x_i(0) - x| + \int _0^t | v_i(s) - u(\eta (x,s),s)|\,\mathrm{d}s. \end{aligned}$$

Here the second term on the right hand side of the above inequality can be estimated as

$$\begin{aligned} \begin{aligned}&\int _0^t | v_i(s) - u(\eta (x,s),s)|\,\mathrm{d}s \\&\quad \leqq \int _0^t | v_i(s) - u(x_i(s),s)|\,\mathrm{d}s + \int _0^t | u(x_i(s),s) - u(\eta (x,s),s)|\,\mathrm{d}s\\&\quad \leqq \int _0^t | v_i(s) - u(x_i(s),s)|\,\mathrm{d}s + \Vert u\Vert _{Lip} \int _0^t | x_i(s) - \eta (x,s)|\,\mathrm{d}s. \end{aligned} \end{aligned}$$

Thus we get

$$\begin{aligned} \begin{aligned} |x_i(t) - \eta (x,t)|&\leqq |x_i(0) - x| +\int _0^t | v_i(s) - u(x_i(s),s)|\,\mathrm{d}s + \Vert u\Vert _{Lip} \int _0^t | x_i(s) - \eta (x,s)|\,\mathrm{d}s, \end{aligned} \end{aligned}$$

and applying Grönwall’s lemma to the above deduces

$$\begin{aligned} |x_i(t) - \eta (x,t)| \leqq C|x_i(0) - x| +C\int _0^t | v_i(s) - u(x_i(s),s)|\,\mathrm{d}s, \end{aligned}$$

where *C* depends only on $\Vert u\Vert _{L^\infty (0,T;Lip)}$ and *T*. In particular, by taking $x = x_i(0)$, we get

2.6 $$\begin{aligned} |x_i(t) - \eta (x_i(0),t)| \leqq C\int _0^t | v_i(s) - u(x_i(s),s)|\,\mathrm{d}s. \end{aligned}$$

Then for any $\phi \in \mathcal {W}^{1,\infty }({\mathbb {R}}^d)$ we use (2.4) to estimate

2.7 $$\begin{aligned} \begin{aligned}&\left| \int _{{\mathbb {R}}^d} \phi (x) (\rho ^N - \rho )\,\mathrm{d}x\right| \\&\quad = \left| \frac{1}{N} \sum _{i=1}^N \phi (x_i(t)) - \int _{{\mathbb {R}}^d}\phi (\eta (x,t))\rho _0\,\mathrm{d}x \right| \\&\quad = \left| \frac{1}{N} \sum _{i=1}^N (\phi (x_i(t)) - \phi (\eta (x_i(0),t))) + \frac{1}{N}\sum _{i=1}^N \phi (\eta (x_i(0),t))- \int _{{\mathbb {R}}^d}\phi (\eta (x,t))\rho _0\,\mathrm{d}x \right| \\&\quad \leqq \frac{1}{N} \sum _{i=1}^N |\phi (x_i(t)) - \phi (\eta (x_i(0),t))| + \left| \frac{1}{N}\sum _{i=1}^N \phi (\eta (x_i(0),t)) - \int _{{\mathbb {R}}^d}\phi (\eta (x,t))\rho _0\,\mathrm{d}x \right| \\&\quad =: L_1 + L_2. \end{aligned} \end{aligned}$$

For $L_1$, we use the Lipschitz continuity together with (2.6) to obtain

2.8 $$\begin{aligned} \begin{aligned} L_1&\leqq \frac{\Vert \phi \Vert _{Lip}}{N}\sum _{i=1}^N |x_i(t) - \eta (x_i(0),t)| \leqq \frac{\Vert \phi \Vert _{Lip}}{N}\int _0^t\sum _{i=1}^N| v_i(s) - u(x_i(s),s)|\,\mathrm{d}s\\&\leqq \Vert \phi \Vert _{Lip}\sqrt{T}\left( \int _0^t \frac{1}{N}\sum _{i=1}^N| v_i(s) - u(x_i(s),s)|^2\,\mathrm{d}s\right) ^{1/2} \\&= \Vert \phi \Vert _{Lip}\sqrt{T}\left( \int _0^t \mathcal {E}^N (\mathcal {Z}^N(s) | U(s))\,\mathrm{d}s \right) ^{1/2}. \end{aligned} \end{aligned}$$

For the estimate of $L_2$, we notice that

$$\begin{aligned} \frac{1}{N}\sum _{i=1}^N \phi (\eta (x_i(0),t)) = \int _{{\mathbb {R}}^d}\phi (\eta (x,t))\rho ^N_0\,\mathrm{d}x. \end{aligned}$$

Using this identity, the Lipschitz estimate for $\eta$ in (2.5), and the fact $\phi \in \mathcal {W}^{1,\infty }({\mathbb {R}}^d)$, we find

2.9 $$\begin{aligned} L_2 = \left| \int _{{\mathbb {R}}^d}\phi (\eta (x,t))(\rho ^N_0 - \rho _0)\,\mathrm{d}x \right| \leqq \left( \Vert \phi \Vert _{L^\infty } + \Vert \phi \Vert _{Lip}\Vert \eta \Vert _{Lip}\right) \,d_{BL}(\rho ^N_0, \rho _0).\nonumber \\ \end{aligned}$$

Putting (2.8) and (2.9) into (2.7) yields

$$\begin{aligned} d_{BL}(\rho ^N_t(\cdot ),\rho (\cdot ,t))\leqq Cd_{BL}(\rho ^N_0, \rho _0) + C\left( \int _0^t \mathcal {E}^N (\mathcal {Z}^N(s) | U(s))\,\mathrm{d}s \right) ^{1/2} \end{aligned}$$

for $0 \leqq t \leqq T$, where $C > 0$ depends only on $\Vert u\Vert _{L^\infty (0,T;Lip)}$ and *T*. $\quad \square$

### Proof of Theorem 1.1

#### Quantitative bound estimates

Applying Grönwall’s lemma and Young’s inequality to the differential inequality in Proposition 2.1 yields

$$\begin{aligned} \mathcal {E}^N (\mathcal {Z}^N(t) | U(t)) \leqq C\mathcal {E}^N (\mathcal {Z}^N_0 | U_0) + C \int _0^t d^2_{BL}(\rho ^N_s(\cdot ),\rho (\cdot ,s))\,\mathrm{d}s, \end{aligned}$$

where $C > 0$ is independent of *N*. We then use Proposition 2.2 to have

$$\begin{aligned} \begin{aligned}&\mathcal {E}^N (\mathcal {Z}^N(t) | U(t)) + d_{BL}^2(\rho ^N_t(\cdot ),\rho (\cdot ,t)) \leqq \, C\mathcal {E}^N (\mathcal {Z}^N_0 | U_0) + Cd_{BL}^2(\rho ^N_0,\rho _0) \\&\quad + C \int _0^t d^2_{BL}(\rho ^N_s(\cdot ),\rho (\cdot ,s))\,\mathrm{d}s + C\int _0^t \mathcal {E}^N (\mathcal {Z}^N(s) | U(s))\,\mathrm{d}s. \end{aligned} \end{aligned}$$

We finally apply Grönwall’s to the above to conclude the desired result.

#### Convergence estimates

For the convergence estimates, it suffices to prove the following lemma:

##### Lemma 2.1

(i) Convergence of local moment:
  $$\begin{aligned} d_{BL}\left( \int _{{\mathbb {R}}^d}v \,\mu ^N(\mathrm{d}v), \, \rho u\right) \leqq \left( \iint _{{\mathbb {R}}^d \times {\mathbb {R}}^d}|v - u(x) |^2\mu ^N(\mathrm{d}x\mathrm{d}v) \right) ^{1/2} + Cd_{BL}(\rho ^N, \rho ). \end{aligned}$$
(ii) Convergence of local energy:
  $$\begin{aligned} \begin{aligned}&d_{BL}\left( \int _{{\mathbb {R}}^d}(v \otimes v)\, \mu ^N(\mathrm{d}v), \,\rho u \otimes u\right) \\&\quad \leqq \iint _{{\mathbb {R}}^d \times {\mathbb {R}}^d}|v - u(x) |^2\mu ^N(\mathrm{d}x\mathrm{d}v) + C\left( \iint _{{\mathbb {R}}^d \times {\mathbb {R}}^d}|v - u(x) |^2\mu ^N(\mathrm{d}x\mathrm{d}v) \right) ^{1/2} \\&\quad + Cd_{BL}(\rho ^N, \rho ). \end{aligned} \end{aligned}$$
(iii) Convergence of empirical measure:
  $$\begin{aligned} d^2_{BL}(\mu ^N, \rho \delta _{u}) \leqq C\iint _{{\mathbb {R}}^d \times {\mathbb {R}}^d}|v - u(x) |^2\,\mu ^N(\mathrm{d}x\mathrm{d}v) + Cd^2_{BL}(\rho ^N, \rho ). \end{aligned}$$

Here $C>0$ is independent of *N*.

##### Proof

(i) For any $\phi \in \mathcal {W}^{1,\infty }({\mathbb {R}}^d)$, we get

$$\begin{aligned} \begin{aligned}&\left| \int _{{\mathbb {R}}^d}\phi (x) \left( \int _{{\mathbb {R}}^d}v\, \mu ^N(x,\mathrm{d}v) - (\rho u)(x) \right) \,\mathrm{d}x \right| \\&\quad = \left| \iint _{{\mathbb {R}}^d \times {\mathbb {R}}^d}\phi (x) (v-u(x))\,\mu ^N(\mathrm{d}x\mathrm{d}v) + \int _{{\mathbb {R}}^d}\phi (x) u(x) (\rho ^N(x) - \rho (x))\,\mathrm{d}x \right| \\&\quad \leqq \Vert \phi \Vert _{L^\infty }\left( \iint _{{\mathbb {R}}^d \times {\mathbb {R}}^d}|v-u(x)|\,\mu ^N(\mathrm{d}x\mathrm{d}v) \right) + \Vert \phi u\Vert _{\mathcal {W}^{1,\infty }} \,d_{BL}(\rho ^N, \rho )\\&\quad \leqq \Vert \phi \Vert _{L^\infty }\left( \iint _{{\mathbb {R}}^d \times {\mathbb {R}}^d}|v-u(x)|^2\,\mu ^N(\mathrm{d}x\mathrm{d}v) \right) ^{1/2} \\&\qquad + \left( \Vert \phi \Vert _{L^\infty } \Vert u\Vert _{L^\infty } + \Vert \phi \Vert _{L^\infty }\Vert u\Vert _{Lip} + \Vert u\Vert _{L^\infty }\Vert \phi \Vert _{Lip} \right) d_{BL}(\rho ^N, \rho ). \end{aligned} \end{aligned}$$

(ii) Adding and subtracting, we notice that

$$\begin{aligned} \begin{aligned}&\int _{{\mathbb {R}}^d}(v \otimes v)\, \mu ^N(\mathrm{d}v) - \rho u\otimes u \\&\quad = \int _{{\mathbb {R}}^d}(v - u) \otimes (v-u)\, \mu ^N(dv) + u \otimes \left( \int _{{\mathbb {R}}^d}v\mu ^N(\mathrm{d}v) - \rho u \right) \\&\qquad + \left( \int _{{\mathbb {R}}^d}v\mu ^N(\mathrm{d}v) - \rho u \right) \otimes u + (\rho - \rho ^N) u\otimes u. \end{aligned} \end{aligned}$$

This yields for $\phi \in \mathcal {W}^{1,\infty }({\mathbb {R}}^d)$

$$\begin{aligned} \begin{aligned}&\left| \int _{{\mathbb {R}}^d}\phi (x)\left( \int _{{\mathbb {R}}^d}(v \otimes v)\, \mu ^N(\mathrm{d}v) - (\rho u)(x)\otimes u(x)\right) dx \right| \\&\quad \leqq \Vert \phi \Vert _{L^\infty } \iint _{{\mathbb {R}}^d \times {\mathbb {R}}^d}|v - u|^2 \, \mu ^N(\mathrm{d}x\mathrm{d}v) + 2\Vert \phi u\Vert _{L^\infty \cap Lip}\,d_{BL}\left( \int _{{\mathbb {R}}^d}v \,\mu ^N(\mathrm{d}v), \ \rho u\right) \\&\qquad + \Vert \phi |u|^2\Vert _{\mathcal {W}^{1,\infty }} \,d_{BL}(\rho ^N, \rho ). \end{aligned} \end{aligned}$$

(iii) For any $\varphi \in \mathcal {W}^{1,\infty }({\mathbb {R}}^d \times {\mathbb {R}}^d)$, we find that

$$\begin{aligned} \begin{aligned}&\left| \iint _{{\mathbb {R}}^d \times {\mathbb {R}}^d}\varphi (x,v) \left( \mu ^N(\mathrm{d}x\mathrm{d}v) - \rho (x)\mathrm{d}x \otimes \delta _{u(x)}(\mathrm{d}v)\right) \right| \\&\quad = \left| \iint _{{\mathbb {R}}^d \times {\mathbb {R}}^d}\varphi (x,v)\, \mu ^N(\mathrm{d}x\mathrm{d}v) - \int _{{\mathbb {R}}^d}\varphi (x,u(x))\rho (x)\,\mathrm{d}x \right| \\&\quad = \left| \iint _{{\mathbb {R}}^d \times {\mathbb {R}}^d}(\varphi (x,v) -\varphi (x,u(x)))\,\mu ^N(\mathrm{d}x\mathrm{d}v)+ \int _{{\mathbb {R}}^d}\varphi (x,u(x))(\rho ^N - \rho )(x)\,\mathrm{d}x\right| \\&\quad \leqq \Vert \varphi \Vert _{Lip}\iint _{{\mathbb {R}}^d \times {\mathbb {R}}^d}|v - u(x)|\,\mu ^N(\mathrm{d}x\mathrm{d}v) + (\Vert \varphi \Vert _{L^\infty } + \Vert \varphi \Vert _{Lip}\Vert u\Vert _{Lip})d_{BL}(\rho ^N, \rho )\\&\quad \leqq C\left( \iint _{{\mathbb {R}}^d \times {\mathbb {R}}^d}|v - u(x)|^2\,\mu ^N(\mathrm{d}x\mathrm{d}v) \right) ^{1/2} + Cd_{BL}(\rho ^N, \rho ). \end{aligned} \end{aligned}$$

$$\square $$

### Singular interaction potential cases: Coulomb and Riesz potentials

In this part, we discuss the singular interaction potentials. Let $d \geqq 1$ and consider a potential $\widetilde{W}$ has the form

2.10 $$\begin{aligned} \widetilde{W}(x) = |x|^{-\alpha } \quad \max \{d-2,0\} \leqq \alpha < d \quad \forall \, d \geqq 1 \end{aligned}$$

or

2.11 $$\begin{aligned} \widetilde{W}(x) = -\log |x| \quad \text{ for } d=1 \hbox { or } 2. \end{aligned}$$

Note that the case $\alpha =d-2$ with $d \geqq 3$ or (2.11) with $d=2$ corresponds to the Coulomb potential, and the other cases are called Riesz potentials. With these types of singular potentials, in a recent work [66], the quantitative mean-field limit from the particle system (1.1) to the pressureless Euler-type system when $\gamma = 0$, $V\equiv 0$ and $\psi \equiv 0$. More precisely, in [66], the following modulated free energy is employed to measure the error between particle and continuum systems:

$$\begin{aligned} \mathcal {F}^N (\mathcal {Z}^N(t) | U(t)):= \frac{1}{2}\iint _{{\mathbb {R}}^d \times {\mathbb {R}}^d \setminus \Delta } \widetilde{W}(x-y)(\rho ^N - \rho )(x)(\rho ^N - \rho )(y)\,\mathrm{d}x\mathrm{d}y, \end{aligned}$$

where $\Delta$ denotes the diagonal in ${\mathbb {R}}^d \times {\mathbb {R}}^d$.

#### Theorem 2.1

Let $T > 0$ and $\mathcal {Z}^N(t) = \{(x_i(t), v_i(t))\}_{i=1}^N$ be a solution to the particle system (1.1), and let $(\rho , u)$ be the unique classical solution of the pressureless Euler system (1.3) with nonlocal interaction forces $\widetilde{W}$, which is appeared in (2.10) or (2.11), instead of *W* up to time $T>0$ with initial data $(\rho _0, u_0)$. Suppose that the communication weight function $\psi$ satisfies $\psi \in \mathcal {W}^{1,\infty }({\mathbb {R}}^d)$. Assume that the classical solution $(\rho ,u)$ satisfies $\rho \in L^\infty (0,T;({\mathcal {P}} \cap L^\infty )({\mathbb {R}}^d))$ and $u \in L^\infty (0,T;\mathcal {W}^{1,\infty }({\mathbb {R}}^d))$. In the case $\alpha \geqq d-1$, we further assume that $\rho \in L^\infty (0,T;{\mathcal {C}}^\sigma ({\mathbb {R}}^d))$ for some $\sigma > \alpha -d+1$. Then there exists $\beta < 2$ such that

2.12 $$\begin{aligned} \begin{aligned}&\iint _{{\mathbb {R}}^d \times {\mathbb {R}}^d}|v - u(x,t) |^2\,\mu ^N_t(\mathrm{d}x\mathrm{d}v) \,+ d^2_{BL}(\rho ^N_t(\cdot ), \rho (\cdot ,t)) \\&\qquad + \iint _{{\mathbb {R}}^d \times {\mathbb {R}}^d \setminus \Delta } \widetilde{W}(x-y)(\rho ^N - \rho )(x)(\rho ^N - \rho )(y)\,\mathrm{d}x\mathrm{d}y \\&\quad \leqq C\iint _{{\mathbb {R}}^d \times {\mathbb {R}}^d}|v - u_0(x) |^2\,\mu ^N_0(\mathrm{d}x\mathrm{d}v) + Cd^2_{BL}(\rho ^N_0, \rho _0) \\&\qquad + C \iint _{{\mathbb {R}}^d \times {\mathbb {R}}^d \setminus \Delta } \widetilde{W}(x-y)(\rho ^N_0 - \rho _0)(x)(\rho ^N_0 - \rho _0)(y)\,\mathrm{d}x\mathrm{d}y + CN^{\beta -2}, \end{aligned} \end{aligned}$$

where $C>0$ is independent of *N*.

#### Remark 2.2

If the interaction potential *W* is singular at the origin, then the term related to *W* in (1.1) should be replaced by $\frac{1}{N} \sum _{j:j \ne i} \nabla _x W (x_i - x_j)$ since *W*(0) can not be well defined. This is why the diagonal $\Delta$ is excluded in the integration in the modulated potential energy.

#### Remark 2.3

If the right hand side of (2.12) converges to zero as $N \rightarrow \infty$, then we also have the same convergence estimates in Theorem 1.1.

#### Remark 2.4

Our quantified mean-field limit estimate from (1.1) to (1.3) also apply with a simple combination of Theorems 1.1 and 2.1 for interaction potentials of the form ${\overline{W}} := W + \widetilde{W}$ with *W* satisfying $\nabla W \in \mathcal {W}^{1,\infty }({\mathbb {R}}^d)$ and $\widetilde{W}$ appeared in (2.10) or (2.11).

#### Proof of Theorem 2.1

For the proof, we only need to reestimate $I_3$ term in the proof of Proposition 2.1. Although this proof is almost the same with that of [66], we provide the details here for the completeness of our work. Let us denote by

$$\begin{aligned} \begin{aligned} I&:= - \frac{1}{N} \sum _{i=1}^N \int _{{\mathbb {R}}^d} (u(x_i(t),t) - v_i(t)) \cdot \nabla _x \widetilde{W}(x_i(t) - y) \rho (y,t)\,\mathrm{d}y\\&\quad + \frac{1}{N^2} \sum _{i \ne j} (u(x_i(t),t) - v_i(t)) \cdot \nabla _x \widetilde{W}(x_i(t) - x_j(t)). \end{aligned} \end{aligned}$$

On the other hand, we find that

$$\begin{aligned} \begin{aligned}&\frac{\mathrm{d}}{\mathrm{d}t}\mathcal {F}^N (\mathcal {Z}^N(t) | U(t)) = \,\frac{1}{2}\frac{\mathrm{d}}{\mathrm{d}t}\left( \frac{1}{N^2} \sum _{i \ne j} \widetilde{W}(x_i - x_j) \right) \\&\qquad - \frac{\mathrm{d}}{\mathrm{d}t}\left( \frac{1}{N} \sum _{i=1}^N \int _{{\mathbb {R}}^d} \widetilde{W}(x_i - y) \rho (y)\,\mathrm{d}y \right) \\&\qquad + \frac{1}{2}\frac{\mathrm{d}}{\mathrm{d}t}\left( \iint _{{\mathbb {R}}^d \times {\mathbb {R}}^d}\widetilde{W}(x - y)\rho (x)\rho (y)\,\mathrm{d}x\mathrm{d}y \right) \\&\quad = \, \frac{1}{N^2} \sum _{i \ne j} \nabla _x \widetilde{W}(x_i - x_j) \cdot v_i - \frac{1}{N} \sum _{i=1}^N \int _{{\mathbb {R}}^d}\nabla _x \widetilde{W}(x_i - y) \cdot v_i \rho (y)\,\mathrm{d}y\\&\qquad - \frac{1}{N}\sum _{i=1}^N \int _{{\mathbb {R}}^d}\nabla _x \widetilde{W}(x_i - y) \cdot (\rho u)(y)\,\mathrm{d}y \\&\qquad + \iint _{{\mathbb {R}}^d \times {\mathbb {R}}^d}\nabla _x \widetilde{W}(x-y) (\rho u)(x) \rho (y)\,\mathrm{d}x\mathrm{d}y. \end{aligned} \end{aligned}$$

Here we used

2.13 $$\begin{aligned} \nabla _x \widetilde{W}(-x) = -\nabla _x \widetilde{W}(x) \quad \text{ for } \quad x \in {\mathbb {R}}^d \setminus \{0\}. \end{aligned}$$

This implies

$$\begin{aligned} \begin{aligned} I&:= - \frac{1}{2}\frac{\mathrm{d}}{\mathrm{d}t} \iint _{{\mathbb {R}}^d \times {\mathbb {R}}^d \setminus \Delta } \widetilde{W}(x-y)(\rho ^N - \rho )(x)(\rho ^N - \rho )(y)\,\mathrm{d}x\mathrm{d}y\\&\quad + \frac{1}{N^2} \sum _{i\ne j} u(x_i) \cdot \nabla _x \widetilde{W}(x_i - x_j) - \frac{1}{N}\sum _{i=1}^N \int _{{\mathbb {R}}^d} \nabla _x \widetilde{W}(x_i -y) \cdot (u(x_i) - u(y)) \rho (y)\,\mathrm{d}y\\&\quad + \iint _{{\mathbb {R}}^d \times {\mathbb {R}}^d} \nabla _x \widetilde{W}(x-y) (\rho u)(x) \rho (y)\,\mathrm{d}x\mathrm{d}y. \end{aligned} \end{aligned}$$

We next use (2.13) to get

$$\begin{aligned} \frac{1}{N^2} \sum _{i\ne j} u(x_i) \cdot \nabla _x \widetilde{W}(x_i - x_j) = \frac{1}{2}\frac{1}{N^2} \sum _{i\ne j} \left( u(x_i) - u(x_j)\right) \cdot \nabla _x \widetilde{W}(x_i - x_j) \end{aligned}$$

and

$$\begin{aligned}&\iint _{{\mathbb {R}}^d \times {\mathbb {R}}^d}\nabla _x \widetilde{W}(x-y) (\rho u)(x) \rho (y)\,\mathrm{d}x\mathrm{d}y \\&\quad = \frac{1}{2}\iint _{{\mathbb {R}}^d \times {\mathbb {R}}^d}\nabla _x \widetilde{W}(x-y) \left( u(x) - u(y)\right) \rho (x) \rho (y)\,\mathrm{d}x\mathrm{d}y. \end{aligned}$$

Thus we obtain

$$\begin{aligned} \begin{aligned} I&:= - \frac{1}{2}\frac{\mathrm{d}}{\mathrm{d}t} \iint _{{\mathbb {R}}^d \times {\mathbb {R}}^d \setminus \Delta } \widetilde{W}(x-y)(\rho ^N - \rho )(x)(\rho ^N - \rho )(y)\,\mathrm{d}x\mathrm{d}y\\&\quad \,\, + \frac{1}{2}\iint _{{\mathbb {R}}^d \times {\mathbb {R}}^d \setminus \Delta } \left( u(x) - u(y) \right) \cdot \nabla _x \widetilde{W} (x-y) (\rho ^N - \rho )(x) (\rho ^N - \rho )(y)\,\mathrm{d}x\mathrm{d}y. \end{aligned} \end{aligned}$$

This together with the estimates in Proposition 2.1 yields

$$\begin{aligned} \begin{aligned}&\frac{\mathrm{d}}{\mathrm{d}t}\left( \mathcal {E}^N (\mathcal {Z}^N(t) | U(t)) \right. \left. +\mathcal {F}^N (\mathcal {Z}^N(t) | U(t))\right) \\&\qquad + 2\gamma \mathcal {E}^N (\mathcal {Z}^N(t) | U(t)) + \frac{1}{N^2}\sum _{i,j=1}^N \psi (x_i - x_j)|v_i - u(x_i)|^2\\&\quad \leqq C\mathcal {E}^N (\mathcal {Z}^N(t) | U(t)) + C d_{BL}^2(\rho ^N,\rho )\\&\qquad + \frac{1}{2}\iint _{{\mathbb {R}}^d \times {\mathbb {R}}^d \setminus \Delta } \left( u(x) - u(y) \right) \cdot \nabla _x \widetilde{W} (x-y) (\rho ^N - \rho )(x) (\rho ^N - \rho )(y)\,\mathrm{d}x\mathrm{d}y. \end{aligned} \end{aligned}$$

We then apply [66, Proposition 1.1] to have that the last term on the right hand side of the above inequality can be bounded from above by

$$\begin{aligned} C\mathcal {F}^N (\mathcal {Z}^N(t) | U(t)) + CN^{\beta -2} \end{aligned}$$

for some $\beta < 2$, where $C>0$ is independent of *N*. Applying the Grönwall’s lemma to the resulting inequality concludes the desired quantitative bound estimate. The convergence result can be directly obtained by using Lemma 2.1. This completes the proof. $\quad \square$

## Combined Small Inertia & Mean Field Limits: From Newton to Aggregation

### Proof of Theorem 1.2

We first start with the case of smooth interaction potentials as in previous section and apply a similar strategy to the proof of Proposition 2.1 to the system (1.16). Then we get

$$\begin{aligned} \frac{\mathrm{d}}{\mathrm{d}t} \mathcal {E}^N (\mathcal {Z}^N(t) | {\bar{U}}(t)) =: \frac{1}{\varepsilon _N}\left( \sum _{i=1}^4 {\bar{I}}_i \right) + {\bar{I}}_5, \end{aligned}$$

where ${\bar{I}}_i, i=1,2,3,4$ are the terms $I_i,i=1,2,3,4$ in (2.2) with replacing $(\rho ,u)$ by $({\bar{\rho }}, {\bar{u}})$, and ${\bar{I}}_5$ is given by

$$\begin{aligned} {\bar{I}}_5 := \frac{1}{N} \sum _{i=1}^N ({\bar{u}}(x_i) - v_i) \cdot {\bar{e}}, \end{aligned}$$

where ${\bar{e}} = \partial _t {\bar{u}} + {\bar{u}} \cdot \nabla _x {\bar{u}}$. This can be simply estimated as

$$\begin{aligned} \begin{aligned}&|{\bar{I}}_5| \leqq \Vert {\bar{e}}\Vert _{L^\infty }\frac{1}{N} \sum _{i=1}^N |{\bar{u}}(x_i) - v_i| \\&\quad \leqq \frac{C}{\varepsilon _N}\frac{1}{N}\sum _{i=1}^N |{\bar{u}}(x_i) - v_i|^2 + C\varepsilon _N \leqq \frac{C}{\varepsilon _N}\mathcal {E}^N (\mathcal {Z}^N(t) | {\bar{U}}(t)) + C\varepsilon _N. \end{aligned} \end{aligned}$$

where $C>0$ depends only $\Vert {\bar{e}}\Vert _{L^\infty }$, independent of *N* and $\varepsilon _N$. For the rest, we employ almost the same arguments as before to have

$$\begin{aligned} \begin{aligned}&\frac{1}{\varepsilon _N}\left( \sum _{i=1}^4 {\bar{I}}_i \right) \leqq - \frac{2\gamma }{\varepsilon _N} \mathcal {E}^N (\mathcal {Z}^N(t) | {\bar{U}}(t))- \frac{1}{\varepsilon _N N^2} \sum _{i,j=1}^N \psi (x_i - x_j)|v_i - {\bar{u}}(x_i)|^2 \\&\quad + \frac{C}{\varepsilon _N}\mathcal {E}^N (\mathcal {Z}^N(t) | {\bar{U}}(t)) + C d_{BL}^2(\rho ^N_t(\cdot ), \rho (\cdot ,t)) , \end{aligned} \end{aligned}$$

where $C>0$ is independent of *N*, $\varepsilon _N$, and $\gamma > 0$. This yields

3.1 $$\begin{aligned} \frac{\mathrm{d}}{\mathrm{d}t} \mathcal {E}^N (\mathcal {Z}^N(t) | {\bar{U}}(t)) + \frac{2\gamma - C}{\varepsilon _N} \mathcal {E}^N (\mathcal {Z}^N(t) | {\bar{U}}(t)) \leqq \frac{C}{\varepsilon _N}d_{BL}^2(\rho ^N_t(\cdot ), \rho (\cdot ,t)) + C\varepsilon _N,\nonumber \\ \end{aligned}$$

where $C>0$ is independent of *N*, $\varepsilon _N$, and $\gamma > 0$. On the other hand, by Proposition 2.2, we can bound the first term on the right hand side of the above inequality from above by

$$\begin{aligned} \frac{C}{\varepsilon _N}d_{BL}^2(\rho ^N_0,{\bar{\rho }}_0) + \frac{C}{\varepsilon _N}\int _0^t \mathcal {E}^N (\mathcal {Z}^N(s) | {\bar{U}}(s))\,\mathrm{d}s, \end{aligned}$$

where $C>0$ is independent of *N*, $\varepsilon _N$, and $\gamma > 0$. This together with integrating (3.1) in time implies

$$\begin{aligned} \begin{aligned}&\mathcal {E}^N (\mathcal {Z}^N(t) | {\bar{U}}(t)) + \frac{2\gamma - C}{\varepsilon _N} \int _0^t \mathcal {E}^N (\mathcal {Z}^N(s) | {\bar{U}}(s))\,\mathrm{d}s \\&\qquad + \frac{1}{\varepsilon _N N^2} \sum _{i,j=1}^N \int _0^t \psi (x_i(s) - x_j(s))|v_i(s) - {\bar{u}}(x_i(s),s)|^2\,\mathrm{d}s \\&\quad \leqq \mathcal {E}^N (\mathcal {Z}^N_0 | {\bar{U}}_0) + \frac{C}{\varepsilon _N}d_{BL}^2(\rho ^N_0,{\bar{\rho }}_0) + C\varepsilon _N. \end{aligned} \end{aligned}$$

We finally apply Grönwall’s lemma to conclude the desired result in Theorem 1.2.

### Singular interaction potential cases

Similarly as before, Theorem 1.2 can be also easily extended to the case with Coulomb or Riesz potentials $\widetilde{W}$ defined in (2.10) or (2.11). More specifically, we have the following theorem.

#### Theorem 3.1

Let $T>0$ and $\mathcal {Z}^N(t) = \{(x_i(t), v_i(t))\}_{i=1}^N$ be a solution to the particle system (1.1), and let $({\bar{\rho }}, {\bar{u}})$ be the unique classical solution of the aggregation-type equation (1.4)–(1.5) with $\widetilde{W}$, which is appeared in (2.10) or (2.11), instead of *W*, under the assumptions of Theorem 1.2 up to time $T>0$ with the initial data ${\bar{\rho }}_0$. Suppose that the strength of damping $\gamma >0$ is large enough and $({\bar{\rho }},{\bar{u}})$ satisfies ${\bar{\rho }} \in L^\infty ({\mathbb {R}}^d \times (0,T))$. We further assume that ${\bar{\rho }} \in L^\infty (0,T;{\mathcal {C}}^\sigma ({\mathbb {R}}^d))$ for some $\sigma > \alpha -d+1$ in the case $s \geqq d-1$. Then there exists $\beta < 2$ such that

$$\begin{aligned} \begin{aligned}&d_{BL}^2(\rho ^N_t(\cdot ),{\bar{\rho }}(\cdot ,t)) + \iint _{{\mathbb {R}}^d \times {\mathbb {R}}^d \setminus \Delta } \,\widetilde{W}(x-y)(\rho ^N - {\bar{\rho }})(x)(\rho ^N - {\bar{\rho }})(y)\,\mathrm{d}x\mathrm{d}y \\&\qquad +\int _0^t \iint _{{\mathbb {R}}^d \times {\mathbb {R}}^d}|v - {\bar{u}}(x,s) |^2\mu ^N_s(\mathrm{d}x\mathrm{d}v) \,\mathrm{d}s\\&\quad \leqq Cd_{BL}^2(\rho ^N_0,{\bar{\rho }}_0) + C \iint _{{\mathbb {R}}^d \times {\mathbb {R}}^d \setminus \Delta } \widetilde{W}(x-y)(\rho ^N_0 - {\bar{\rho }}_0)(x)(\rho ^N_0 - {\bar{\rho }}_0)(y)\,\mathrm{d}x\mathrm{d}y \\&\qquad + C\varepsilon _N\iint _{{\mathbb {R}}^d \times {\mathbb {R}}^d}|v - {\bar{u}}_0(x) |^2\mu ^N_0(\mathrm{d}x\mathrm{d}v) + C\varepsilon _N^2 + CN^{\beta -2} \\ \end{aligned} \end{aligned}$$

and

$$\begin{aligned} \begin{aligned}&\frac{1}{\varepsilon _N}d^2_{BL}(\rho ^N_t(\cdot ),{\bar{\rho }}(\cdot ,t)) + \frac{1}{\varepsilon _N}\,\iint _{{\mathbb {R}}^d \times {\mathbb {R}}^d \setminus \Delta } \widetilde{W}(x-y)(\rho ^N - {\bar{\rho }})(x)(\rho ^N - {\bar{\rho }})(y)\,\mathrm{d}x\mathrm{d}y \\&\qquad + \iint _{{\mathbb {R}}^d \times {\mathbb {R}}^d}|v - {\bar{u}}(x,t) |^2\mu ^N_t(\mathrm{d}x\mathrm{d}v) \\&\quad \leqq \frac{C}{\varepsilon _N}d_{BL}^2(\rho ^N_0,{\bar{\rho }}_0) + \frac{C}{\varepsilon _N} \iint _{{\mathbb {R}}^d \times {\mathbb {R}}^d \setminus \Delta } \widetilde{W}(x-y)(\rho ^N_0 - {\bar{\rho }}_0)(x)(\rho ^N_0 - {\bar{\rho }}_0)(y)\,\mathrm{d}x\mathrm{d}y \\&\qquad + C(1 + \varepsilon _N)\iint _{{\mathbb {R}}^d \times {\mathbb {R}}^d}|v - {\bar{u}}_0(x) |^2\mu ^N_0(\mathrm{d}x\mathrm{d}v) + C\varepsilon _N + C\frac{N^{\beta -2}}{\varepsilon _N} \end{aligned} \end{aligned}$$

for all $t \in [0,T]$, where $C>0$ is independent of $\varepsilon _N$ and *N*. In particular if

$$\begin{aligned} \iint _{{\mathbb {R}}^d \times {\mathbb {R}}^d}|v - {\bar{u}}_0(x) |^2\mu ^N_0(\mathrm{d}x\mathrm{d}v) \leqq C\varepsilon _N \end{aligned}$$

and

$$\begin{aligned} d^2_{BL}(\rho ^N_0,{\bar{\rho }}_0) + \iint _{{\mathbb {R}}^d \times {\mathbb {R}}^d \setminus \Delta } \widetilde{W}(x-y)(\rho ^N_0 - {\bar{\rho }}_0)(x)(\rho ^N_0 - {\bar{\rho }}_0)(y)\,\mathrm{d}x\mathrm{d}y \leqq C\varepsilon _N^2 \end{aligned}$$

for some $C>0$ which is independent of $\varepsilon _N$, then we have

$$\begin{aligned} \begin{aligned}&d_{BL}^2(\rho ^N_t(\cdot ),{\bar{\rho }}(\cdot ,t)) + \iint _{{\mathbb {R}}^d \times {\mathbb {R}}^d \setminus \Delta } \widetilde{W}(x-y)(\rho ^N - {\bar{\rho }})(x)(\rho ^N - {\bar{\rho }})(y)\,\mathrm{d}x\mathrm{d}y \\&\quad \leqq C\varepsilon _N^2 + CN^{\beta -2} \end{aligned} \end{aligned}$$

and

$$\begin{aligned} \iint _{{\mathbb {R}}^d \times {\mathbb {R}}^d}|v - {\bar{u}}(x,t) |^2\mu ^N_t(\mathrm{d}x\mathrm{d}v) \leqq C\varepsilon _N + C\frac{N^{\beta -2}}{\varepsilon _N} \end{aligned}$$

for all $t \in [0,T]$, where $C>0$ is independent of $\varepsilon _N$ and *N*.

## Local Cauchy Problem for Pressureless Euler Equations with Nonlocal Forces

In order to make the analysis for the mean-field limit from the particle system (1.1) to the pressureless Euler-type equations (1.3) fully rigorous, we need to have the existence of solutions for both systems. As mentioned in Introduction, we postpone the existence theory for the particle system (1.1) in Appendix A, and here we provide local-in-time existence and uniqueness of classical solutions for the system (1.3). For the reader’s convenience, let us recall our limiting system:

4.1 $$\begin{aligned} \begin{aligned}&\partial _t \rho + \nabla _x \cdot (\rho u) = 0, \quad (x,t) \in {\mathbb {R}}^d \times {\mathbb {R}}_+,\\&\quad \partial _t (\rho u) + \nabla _x \cdot (\rho u \otimes u) = -\rho u - \rho \nabla _x V - \rho \nabla _x W \star \rho \\&\quad + \rho \int _{{\mathbb {R}}^d} \psi (x-y) (u(y) - u(x))\,\rho (y)\,\mathrm{d}y, \end{aligned} \end{aligned}$$

with the initial data

$$\begin{aligned} (\rho (x,t),u(x,t))|_{t=0} =: (\rho _0(x), u_0(x)), \quad x \in {\mathbb {R}}^d. \end{aligned}$$

Here we set the coefficient of linear damping $\gamma =1$.

For the one dimensional problem, the well-posedness and singularity formation for the system (4.1) without the linear damping, the confinement and interaction potentials, called Euler-alignment system, are discussed in [13]. To be more precise, the sharp critical threshold which distinguishes the global-in-time regularity of classical solutions and finite-time breakdown of smoothness is analyzed. The sharp critical threshold estimate is also obtained in [15] for the pressureless damped Euler–Poisson system with quadratic confinement potential in one dimension, that is the system (4.1) with replacing *W* by ${\mathcal {N}}$, $V = |x|^2/2$, and $\psi \equiv 0$. For the pressureless Euler–Poisson system, the critical threshold is also discussed in [2, 38], see also [69] for the case with pressure. More recently, in [27], the local-in-time existence of classical solutions and finite-time singularity formation are taken into account.

We introduce the exact notion of strong solution to the system (4.1) that we will deal with.

### Definition 4.1

Let $s > d/2+1$. For given $T\in (0,\infty )$, the pair $(\rho ,u)$ is a strong solution of (4.1) on the time interval [0, *T*] if and only if the following conditions are satisfied:

(i) $$\rho \in {\mathcal {C}}([0,T];H^s({\mathbb {R}}^d))$$, $$u \in {\mathcal {C}}([0,T];Lip({\mathbb {R}}^d)\cap L^2_{loc}({\mathbb {R}}^d))$$, and $$\nabla _x^2 u \in {\mathcal {C}}([0,T];H^{s-1}({\mathbb {R}}^d))$$,
(ii) $(\rho , u)$ satisfy the system (4.1) in the sense of distributions.

Notice that due to the choice of *s* in the previous definition, these strong solutions are also classical solutions to (4.1). Our main result of this section is the following local Cauchy problem for the system (4.1).

### Theorem 4.1

Let $s > d/2+1$ and $R>0$. Suppose that the confinement potential *V* is given by $V = |x|^2/2$, the interaction potential $\nabla _x W \in (\mathcal {W}^{1,1} \cap \mathcal {W}^{1,\infty })({\mathbb {R}}^d)$, and the communication weight function $\psi$ satisfies

4.2 $$\begin{aligned} \psi \in {\mathcal {C}}_c^1({\mathbb {R}}^d) \quad \text{ and } \quad supp(\psi ) \subseteq B(0,R), \end{aligned}$$

where $B(0,R) \subset {\mathbb {R}}^d$ denotes a ball of radius *R* centered at the origin. For any $N<M$, there is a positive constant $T^*$ depending only on *R*, *N*, and *M* such that if $\rho _0 > 0$ on ${\mathbb {R}}^d$ and

$$\begin{aligned} \Vert \rho _0\Vert _{H^s} + \Vert u_0\Vert _{L^2(B(0,R))}+\Vert \nabla _x u_0\Vert _{L^\infty } + \Vert \nabla _x^2 u_0\Vert _{H^{s-1}} < N, \end{aligned}$$

then the Cauchy problem (4.1) has a unique strong solution $(\rho ,u)$, in the sense of Definition 4.1, satisfying

$$\begin{aligned} \sup _{0 \leqq t \leqq T^*}\left( \Vert \rho (\cdot ,t)\Vert _{H^s} + \Vert u(\cdot ,t)\Vert _{L^2(B(0,R))} +\Vert \nabla _x u(\cdot ,t)\Vert _{L^\infty } + \Vert \nabla _x^2 u(\cdot ,t)\Vert _{H^{s-1}}\right) \leqq M. \end{aligned}$$

### Remark 4.1

The assumption on the communication weight function (4.2) implies $\psi \in \mathcal {W}^{1,p}({\mathbb {R}}^d)$ for any $p \in [1,\infty ]$.

### Remark 4.2

By the standard Sobolev embedding theorem, the solution $(\rho ,u)$ constructed as in Theorem 4.1 is a classical solution, that is $(\rho ,u) \in {\mathcal {C}}^1({\mathbb {R}}^d \times (0,T^*))$.

### Remark 4.3

The $L^2$-norm of *u* on the ball is introduced due to the confinement potential *V*. In fact, if we ignore the confinement potential *V* in the momentum equation in (4.1), then under the following assumption on the initial data

$$\begin{aligned} \Vert \rho _0\Vert _{H^s} + \Vert u_0\Vert _{H^{s+1}} < N, \end{aligned}$$

we have the unique strong solution $(\rho ,u)$ to the system (4.1) satisfying

$$\begin{aligned} \sup _{0 \leqq t \leqq T^*}\left( \Vert \rho (\cdot ,t)\Vert _{H^s} + \Vert u(\cdot ,t)\Vert _{H^{s+1}}\right) \leqq M. \end{aligned}$$

### Remark 4.4

In case of a singular interaction potential beyond the Coulomb case, we refer to [27] for the well-posedness theory for the Euler–Riesz system. More precisely, in [27], the local-in-time existence and uniqueness of classical solutions to the system (4.1) with ${\widetilde{W}}$ defined in (2.10) instead of the regular *W*, $\gamma =0$, $V \equiv 0$, and $\psi \equiv 0$ are discussed. One may extend the arguments used in [27] to study the well-posedness for the system (4.1) with ${\widetilde{W}}$.

### Linearized system

In this part, we construct approximate solutions $(\rho ^n, u^n)$ for the system (4.1) and provide some uniform bound estimates of it.

Let us first take the initial data as the zeroth approximation:

$$\begin{aligned} (\rho ^0(x,t),u^0(x,t)) = (\rho _0(x),u_0(x)), \quad (x,t) \in {\mathbb {R}}^d \times {\mathbb {R}}_+. \end{aligned}$$

We next suppose that the *n*th approximation $(\rho ^n, u^n)$ with $n \geqq 1$ is given. Then we define the $(n+1)$th approximation $(\rho ^{n+1}, u^{n+1})$ as a solution to the following linear system.

4.3 $$\begin{aligned}&\partial _t \rho ^{n+1} + u^n \cdot \nabla \rho ^{n+1} + \rho ^{n+1} \nabla \cdot u^n = 0, \quad (x,t) \in {\mathbb {R}}^d \times {\mathbb {R}}_+,\nonumber \\&\quad \rho ^{n+1}\partial _t u^{n+1} + \rho ^{n+1} u^n \cdot \nabla u^{n+1} = - \rho ^{n+1}u^{n+1} - \rho ^{n+1}(\nabla _x V + \nabla _x W \star \rho ^{n+1})\nonumber \\&\quad + \rho ^{n +1} \int _{{\mathbb {R}}^d}\psi (x-y) (u^n(y) - u^n(x)) \rho ^{n+1}(y)\,\mathrm{d}y, \end{aligned}$$

with the initial data

$$\begin{aligned} (\rho ^n(x,0),u^n(x,0))=(\rho _0(x),u_0(x)) \quad \text{ for } \text{ all } \quad n \geqq 1, \quad x\in {\mathbb {R}}^d. \end{aligned}$$

Let us introduce a solution space ${\mathcal {Y}}_{s,R}(T)$ with $s > d/2+1$ as

$$\begin{aligned} \begin{aligned}&{\mathcal {Y}}_{s,R}(T) := \Big \{ (\rho ,u) : \rho \in {\mathcal {C}}([0,T];H^s({\mathbb {R}}^d)), u \in {\mathcal {C}}([0,T]; \\&\quad L^2(B(0,R))) \cap {\mathcal {C}}([0,T];{\dot{\mathcal {W}}}^{1,\infty }({\mathbb {R}}^d)), \\&\quad \nabla _x^2 u \in {\mathcal {C}}([0,T];H^{s-1}({\mathbb {R}}^d)) \Big \}. \end{aligned} \end{aligned}$$

Then by the standard linear solvability theory [58], for any $T>0$ we have that the approximation $\{(\rho ^n,u^n)\}_{n=0}^\infty \subset {\mathcal {Y}}_{s,R}(T)$ is well-defined.

For notational simplicity, in the rest of this section, we drop *x*-dependence of the differential operator $\nabla _x$.

#### Proposition 4.1

Suppose that the initial data $(\rho _0, u_0)$ satisfies $\rho _0 > 0$ on ${\mathbb {R}}^d$ and

$$\begin{aligned} \Vert \rho _0\Vert _{H^s} + \Vert u_0\Vert _{L^2(B(0,R))} + \Vert \nabla u_0\Vert _{L^\infty } + \Vert \nabla ^2 u_0\Vert _{H^{s-1}} < N, \end{aligned}$$

and let $(\rho ^n,u^n)$ be a sequence of the approximate solutions of (4.3) with the initial data $(\rho _0, u_0)$. Then for any $N < M$, there exists $T^* > 0$ such that

$$\begin{aligned}&\sup _{n \geqq 0} \sup _{0 \leqq t \leqq T^*}\left( \Vert \rho ^n(\cdot ,t)\Vert _{H^s} + \Vert u^n(\cdot ,t)\Vert _{L^2(B(0,R))} \right. \\&\left. \quad + \Vert \nabla u^n(\cdot ,t)\Vert _{L^\infty } + \Vert \nabla ^2 u^n(\cdot ,t)\Vert _{H^{s-1}}\right) \leqq M. \end{aligned}$$

#### Proof

For the proof, we use the inductive argument. Since we take the initial data for the first iteration step, it is clear to find

$$\begin{aligned} \begin{aligned}&\sup _{0 \leqq t \leqq T}\left( \Vert \rho ^0(\cdot ,t)\Vert _{H^s} + \Vert u^0(\cdot ,t)\Vert _{L^2(B(0,R))} + \Vert \nabla u^0(\cdot ,t)\Vert _{L^\infty } + \Vert \nabla ^2 u^0(\cdot ,t)\Vert _{H^{s-1}}\right) \\&\quad = \Vert \rho _0\Vert _{H^s} + \Vert u_0\Vert _{L^2(B(0,R))} + \Vert \nabla u_0\Vert _{L^\infty } + \Vert \nabla ^2 u_0\Vert _{H^{s-1}}< N< M. \end{aligned} \end{aligned}$$

We now suppose that

$$\begin{aligned} \sup _{0 \leqq t \leqq T_0}\left( \Vert \rho ^n(\cdot ,t)\Vert _{H^s} + \Vert u^n(\cdot ,t)\Vert _{L^2(B(0,R))} + \Vert \nabla u^n(\cdot ,t)\Vert _{L^\infty } + \Vert \nabla ^2 u^n(\cdot ,t)\Vert _{H^{s-1}}\right) \leqq M \end{aligned}$$

for some $T_0 > 0$. In the rest of the proof, upon mollifying if necessary we may assume that the communication weight function $\psi$ is smooth. Since this proof is a rather lengthy, we divide it into four steps:

- In **Step A**, we provide the positivity and $H^s({\mathbb {R}}^d)$-estimate of $\rho ^{n+1}$:
  $$\begin{aligned} \rho ^{n+1}(x,t) > 0 \quad \forall \, (x,t) \in {\mathbb {R}}^d \times [0,T] \quad \text{ and } \quad \Vert \rho ^{n+1}(\cdot ,t)\Vert _{H^s} \leqq \Vert \rho _0\Vert _{H^s}e^{CMt} \end{aligned}$$
  for $t \leqq T_0$, where $C>0$ is independent of *n*.
- In **Step B**, we show ${{\dot{\mathcal {W}}}}^{1,\infty }({\mathbb {R}}^d)$-estimate and $L^2(B(0,R))$-estimate of $u^{n+1}$:
  $$\begin{aligned} \begin{aligned}&\Vert \nabla u^{n+1}(\cdot ,t)\Vert _{L^\infty } + \Vert u^{n+1}(\cdot ,t)\Vert _{L^2(B(0,R))} \\&\quad \leqq \Vert \nabla u_0\Vert _{L^\infty }e^{(CM - 1)t} + \Vert u_0\Vert _{L^2(B(0,R))} + E(t) \end{aligned} \end{aligned}$$
  for $t \leqq T_0$, where $C>0$ is independent of *n*, and $E: [0,T_0] \rightarrow [0,\infty )$ is continuous on $[0,T_0]$ satisfying $E(t) \rightarrow 0$ as $t \rightarrow 0^+$.
- In **Step C**, we estimate the higher order derivative of $u^{n+1}$:
  $$\begin{aligned} \Vert \nabla ^2 u^{n+1}\Vert _{H^{s-1}} \leqq \Vert \nabla ^2 u_0\Vert _{H^{s-1}} e^{CMt} + E(t) \end{aligned}$$
  for $t \leqq T_0$, where $C>0$ is independent of *n*, and *E* satisfies the same property as in **Step B**.
- In **Step D**, we finally combine all of the estimates in **Steps A, B, & C** to conclude our desired result.

**Step A.-** We first show the positivity of $\rho ^{n+1}$. Consider the following characteristic flow $\eta ^{n+1}$ associated to the fluid velocity $u^n$ by

4.4 $$\begin{aligned} \partial _t \eta ^{n+1}(x,t) = u^n(\eta ^{n+1}(x,t),t) \quad \text{ for } \quad t > 0 \end{aligned}$$

with the initial data $\eta ^{n+1}(x,0) =x \in {\mathbb {R}}^d.$ Since $u^n$ is globally Lipschitz, the characteristic equations (4.4) are well-defined. Then by using the method of characteristics, we obtain

$$\begin{aligned} \partial _t \rho ^{n+1}(\eta ^{n+1}(x,t),t) = - \rho ^{n+1}(\eta ^{n+1}(x,t),t) (\nabla \cdot u^n)(\eta ^{n+1}(x,t),t), \end{aligned}$$

and applying Grönwall’s lemma yields

$$\begin{aligned} \rho ^{n+1}(\eta ^{n+1}(x,t),t) = \rho _0(x) \exp \left( -\int _0^t (\nabla \cdot u^n)(\eta ^{n+1}(x,\tau ),\tau )\,\mathrm{d}\tau \right) \geqq \rho _0(x) e^{-MT_0} > 0. \end{aligned}$$

We next estimate $H^s$-norm of $\rho ^{n+1}$. We first easily find

$$\begin{aligned} \begin{aligned}&\frac{\mathrm{d}}{\mathrm{d}t}\Vert \rho ^{n+1}\Vert _{L^2}^2 \leqq C\Vert \nabla u^n\Vert _{L^\infty }\Vert \rho ^{n+1}\Vert _{L^2}^2\leqq CM\Vert \rho ^{n+1}\Vert _{L^2}^2,\\&\frac{\mathrm{d}}{\mathrm{d}t}\Vert \nabla \rho ^{n+1}\Vert _{L^2}^2 \leqq C\Vert \nabla u^n\Vert _{L^\infty }\Vert \nabla \rho ^{n+1}\Vert _{L^2}^2 \\&\quad + C\Vert \nabla ^2 u^n\Vert _{L^2}\Vert \rho ^{n+1}\Vert _{L^\infty }\Vert \nabla \rho ^{n+1}\Vert _{L^2} \leqq CM\Vert \rho ^{n+1}\Vert _{H^s}\Vert \nabla \rho ^{n+1}\Vert _{L^2}, \end{aligned} \end{aligned}$$

and

$$\begin{aligned} \begin{aligned}&\frac{1}{2}\frac{\mathrm{d}}{\mathrm{d}t}\int _{{\mathbb {R}}^d} |\nabla ^k \rho ^{n+1}|^2\,\mathrm{d}x \\&\quad = - \int _{{\mathbb {R}}^d} \nabla ^k \rho ^{n+1} \cdot (u^n \cdot \nabla ^{k+1} \rho ^{n+1})\,\mathrm{d}x \\&\qquad - \int _{{\mathbb {R}}^d} \nabla ^k \rho ^{n+1} \cdot (\nabla ^k (\nabla \rho ^{n+1} \cdot u^n) - u^n \cdot \nabla ^{k+1} \rho ^{n+1})\,\mathrm{d}x\\&\qquad - \int _{{\mathbb {R}}^d} \nabla \rho ^{n+1} \cdot (\nabla ^k (\nabla \cdot u^n)) \rho ^{n+1}\,\mathrm{d}x \\&\qquad - \int _{{\mathbb {R}}^d} \nabla ^k \rho ^{n+1} \cdot (\nabla ^k(\rho ^{n+1} \nabla \cdot u^n) - \rho \nabla ^k (\nabla \cdot u^n))\,\mathrm{d}x\\&\quad =: \sum _{i=1}^4 I_i \end{aligned} \end{aligned}$$

for $2 \leqq k \leqq s$. Here we use Moser-type inequality to estimate $I_i,i=1,\cdots ,4$ as

$$\begin{aligned} \begin{aligned} I_1&\leqq \Vert \nabla u^n\Vert _{L^\infty }\Vert \nabla ^k \rho ^{n+1}\Vert _{L^2}^2\leqq CM\Vert \nabla ^k \rho ^{n+1}\Vert _{L^2}^2,\\ I_2&\leqq \Vert \nabla ^k (\nabla \rho ^{n+1} \cdot u^n) - u^n \cdot \nabla ^{k+1} \rho ^{n+1}\Vert _{L^2}\Vert \nabla ^k\rho ^{n+1}\Vert _{L^2}\\&\leqq C\left( \Vert \nabla ^k u^n\Vert _{L^2}\Vert \nabla \rho ^{n+1}\Vert _{L^\infty } + \Vert \nabla u^n\Vert _{L^\infty }\Vert \nabla ^k\rho ^{n+1}\Vert _{L^2}\right) \Vert \nabla ^k\rho ^{n+1}\Vert _{L^2}\\&\leqq CM\Vert \nabla \rho ^{n+1}\Vert _{H^{s-1}}\Vert \nabla ^k\rho ^{n+1}\Vert _{L^2},\\ I_3&\leqq \Vert \rho ^{n+1}\Vert _{L^\infty }\Vert \nabla ^k\rho ^{n+1}\Vert _{L^2}\Vert \nabla ^{k+1} u^n\Vert _{L^2}\leqq CM\Vert \rho ^{n+1}\Vert _{H^s}\Vert \nabla ^k\rho ^{n+1}\Vert _{L^2},\\ I_4&\leqq \Vert \nabla ^k(\rho ^{n+1} \nabla \cdot u^n) - \rho ^{n+1}\nabla ^k (\nabla \cdot u^n)\Vert _{L^2}\Vert \nabla ^k\rho ^{n+1}\Vert _{L^2}\\&\leqq C\left( \Vert \nabla ^k \rho ^{n+1}\Vert _{L^2}\Vert \nabla u^n\Vert _{L^\infty } + \Vert \nabla \rho ^{n+1}\Vert _{L^\infty }\Vert \nabla ^k u^n\Vert _{L^2} \right) \Vert \nabla ^k\rho ^{n+1}\Vert _{L^2}\\&\leqq CM\Vert \nabla \rho ^{n+1}\Vert _{H^{s-1}}\Vert \nabla ^k\rho ^{n+1}\Vert _{L^2}. \end{aligned} \end{aligned}$$

Combining all of the above estimates entails

4.5 $$\begin{aligned} \frac{\mathrm{d}}{\mathrm{d}t}\Vert \rho ^{n+1}\Vert _{H^s} \leqq CM\Vert \rho ^{n+1}\Vert _{H^s}, \quad \text{ that } \text{ is } \quad \Vert \rho ^{n+1}(\cdot ,t)\Vert _{H^s} \leqq \Vert \rho _0\Vert _{H^s}e^{CMt}\nonumber \\ \end{aligned}$$

for $t \leqq T_0$, where $C > 0$ is independent of *n*.

**Step B.-** Due to the positivity of $\rho ^{n+1}$, it follows from the momentum equation in (4.3) that $u^{n+1}$ satisfies

4.6 $$\begin{aligned} \begin{aligned}&\partial _t u^{n+1} + u^n \cdot \nabla u^{n+1} = - u^{n+1} - \nabla V - \nabla W \star \rho ^{n+1} \\&\quad + \int _{{\mathbb {R}}^d}\psi (x-y) (u^n(y) - u^n(x)) \rho ^{n+1}(y)\,\mathrm{d}y. \end{aligned} \end{aligned}$$

Taking the differential operator $\nabla$ to (4.6) gives

4.7 $$\begin{aligned} \partial _t \nabla u^{n+1} + u^n \cdot \nabla ^2 u^{n+1}&= - \nabla u^n \,\nabla u^{n+1} - \nabla u^{n+1} - {\mathbb {I}}_d - \nabla W \star \nabla \rho ^{n+1}\nonumber \\&\quad + \int _{{\mathbb {R}}^d}(u^n(y) - u^n(x)) \otimes \nabla _x \psi (x-y) \rho ^{n+1}(y)\,\mathrm{d}y \nonumber \\&\quad - \nabla u^n \int _{{\mathbb {R}}^d}\psi (x-y) \rho ^{n+1}(y)\,\mathrm{d}y, \end{aligned}$$

where we used $\nabla V = x$ and ${\mathbb {I}}_d$ denotes the $n \times n$ identity matrix. Note that

$$\begin{aligned} |\nabla u^n \,\nabla u^{n+1}| \leqq M\Vert \nabla u^{n+1}(\cdot ,t)\Vert _{L^\infty } \end{aligned}$$

and

$$\begin{aligned} \Vert \nabla W \star \nabla \rho ^{n+1}\Vert _{L^\infty } \leqq \Vert \nabla W\Vert _{L^2} \Vert \nabla \rho ^{n+1}\Vert _{L^2}. \end{aligned}$$

We also estimate the last terms on the right hand side of (4.7) as

$$\begin{aligned} \begin{aligned}&\left| \int _{{\mathbb {R}}^d}(u^n(y) - u^n(x)) \otimes \nabla _x \psi (x-y) \rho ^{n+1}(y)\,\mathrm{d}y \right| \\&\quad \leqq \int _{|x-y| \leqq R} |u^n(y) - u^n(x)| |\nabla _x \psi (x-y)| \rho ^{n+1}(y)\,\mathrm{d}y \\&\quad \leqq \Vert \nabla u^n\Vert _{L^\infty } \int _{|x-y| \leqq R} |y-x| |\nabla _x \psi (x-y)| \rho ^{n+1}(y)\,\mathrm{d}y\\&\quad \leqq \Vert \nabla u^n\Vert _{L^\infty } R \Vert \nabla \psi \Vert _{L^2}\Vert \rho ^{n+1}\Vert _{L^2}\\&\quad \leqq CM\Vert \nabla \psi \Vert _{L^2}\Vert \rho ^{n+1}\Vert _{L^2} \end{aligned} \end{aligned}$$

and

$$\begin{aligned} \left| \nabla u^n \int _{{\mathbb {R}}^d}\psi (x-y) \rho ^{n+1}(y)\,\mathrm{d}y \right| \leqq \Vert \nabla u^n\Vert _{L^\infty } \Vert \psi \Vert _{L^2}\Vert \rho ^{n+1}\Vert _{L^2} \leqq CM\Vert \psi \Vert _{L^2}\Vert \rho ^{n+1}\Vert _{L^2}. \end{aligned}$$

These estimates together with integrating (4.7) along the characteristic flow $\eta ^{n+1}$ implies

$$\begin{aligned} \begin{aligned}&e^t\Vert \nabla u^{n+1}(\cdot ,t)\Vert _{L^\infty } \leqq \Vert \nabla u_0\Vert _{L^\infty } + CM\int _0^t e^\tau \Vert \nabla u^{n+1}(\cdot ,\tau )\Vert _{L^\infty }\,\mathrm{d}\tau \\&\quad + C(1+M)\int _0^t e^\tau \Vert \rho ^{n+1}(\cdot ,\tau )\Vert _{H^s}\,\mathrm{d}\tau . \end{aligned} \end{aligned}$$

By using Grönwall’s lemma, we obtain

$$\begin{aligned} \begin{aligned} e^t\Vert \nabla u^{n+1}(\cdot ,t)\Vert _{L^\infty }&\leqq \Vert \nabla u_0\Vert _{L^\infty }e^{CMt} + C(1+M)\int _0^t e^\tau \Vert \rho ^{n+1}(\cdot ,\tau )\Vert _{H^s}\,\mathrm{d}\tau \\&\quad + CM(1+M) e^{CMt}\int _0^t e^{-CM\xi }\int _0^\xi e^\tau \Vert \rho ^{n+1}(\cdot ,\tau )\Vert _{H^s}\,\mathrm{d}\tau \mathrm{d}\xi . \end{aligned} \end{aligned}$$

This together with (4.5) asserts

4.8 $$\begin{aligned} \Vert \nabla u^{n+1}(\cdot ,t)\Vert _{L^\infty } \leqq \Vert \nabla u_0\Vert _{L^\infty }e^{(CM - 1)t} + E_1(t), \end{aligned}$$

where $E_1: [0,T_0] \rightarrow [0,\infty )$ is continuous on $[0,T_0]$ satisfying $E_1(t) \rightarrow 0$ as $t \rightarrow 0^+$.

For the $L^2$-estimate of $u^{n+1}$ on *B*(0, *R*), we multiply (4.6) by $u^{n+1}$ and integrate it over *B*(0, *R*) to yield

$$\begin{aligned} \begin{aligned}&\frac{1}{2}\frac{\mathrm{d}}{\mathrm{d}t}\int _{B(0,R)}|u^{n+1}|^2\,\mathrm{d}x \\&\quad = \int _{B(0,R)}u^{n+1} \cdot \left( - u^n \cdot \nabla u^{n+1} - u^{n+1} - \nabla V - \nabla W \star \rho ^{n+1}\right) \,\mathrm{d}x \\&\qquad + \int _{B(0,R)}u^{n+1} \cdot \left( \int _{{\mathbb {R}}^d}\psi (x-y) (u^n(y) - u^n(x)) \rho ^{n+1}(y)\,\mathrm{d}y\right) \,\mathrm{d}x\\&\quad \leqq \Vert \nabla u^{n+1}\Vert _{L^\infty } \Vert u^n\Vert _{L^2(B(0,R))}\Vert u^{n+1}\Vert _{L^2(B(0,R))} - \Vert u^{n+1}\Vert _{L^2(B(0,R))}^2\\&\qquad + R\Vert u^{n+1}\Vert _{L^1(B(0,R))} + C(\Vert \rho ^{n+1}\Vert _{L^2} + \Vert \rho ^{n+1}\Vert _{L^\infty }) \Vert u^{n+1}\Vert _{L^1(B(0,R))}\\&\qquad +\Vert \nabla u^n\Vert _{L^\infty } R \Vert \psi \Vert _{L^2}\Vert \rho ^{n+1}\Vert _{L^2} \Vert u^{n+1}\Vert _{L^1(B(0,R))}. \end{aligned} \end{aligned}$$

Here we used

$$\begin{aligned} \begin{aligned}&\left| \int _{{\mathbb {R}}^d}\psi (x-y) (u^n(y) - u^n(x)) \rho ^{n+1}(y)\,\mathrm{d}y\right| \\&\quad \leqq \int _{|x-y| \leqq R} \psi (x-y) |u^n(y) - u^n(x)| \rho ^{n+1}(y)\,\mathrm{d}y\\&\quad \leqq \Vert \nabla u^n\Vert _{L^\infty }\int _{|x-y| \leqq R} \psi (x-y) |x-y| \rho ^{n+1}(y)\,\mathrm{d}y\\&\quad \leqq \Vert \nabla u^n\Vert _{L^\infty } R \Vert \psi \Vert _{L^2}\Vert \rho ^{n+1}\Vert _{L^2}. \end{aligned} \end{aligned}$$

Thus we obtain

$$\begin{aligned} \frac{\mathrm{d}}{\mathrm{d}t}\Vert u^{n+1}\Vert _{L^2(B(0,R))} \leqq CM\Vert \nabla u^{n+1}\Vert _{L^\infty } + C(1 + (1+M)\Vert \rho ^{n+1}\Vert _{H^s} ), \end{aligned}$$

where $C>0$ depends only on *R* and $\Vert \psi \Vert _{L^2}$. Integrating this over [0, *t*] with $t \leqq T_0$ and using the estimates (4.5) and (4.8) imply

4.9 $$\begin{aligned} \Vert u^{n+1}\Vert _{L^2(B(0,R))} \leqq \Vert u_0\Vert _{L^2(B(0,R))} + E_2(t), \end{aligned}$$

where $E_2: [0,T_0] \rightarrow [0,\infty )$ is continuous on $[0,T_0]$ satisfying $E_2(t) \rightarrow 0$ as $t \rightarrow 0^+$.

**Step C.-** For $2 \leqq k \leqq s+1$, we find

$$\begin{aligned} \begin{aligned}&\frac{1}{2}\frac{\mathrm{d}}{\mathrm{d}t}\int _{{\mathbb {R}}^d} |\nabla ^k u^{n+1}|^2\,\mathrm{d}x \\&\quad = - \int _{{\mathbb {R}}^d} \nabla ^k u^{n+1} \cdot (u^n \cdot \nabla ^{k+1} u^{n+1})\,\mathrm{d}x \\&\qquad - \int _{{\mathbb {R}}^d} \nabla ^k u \cdot ( \nabla ^k(u^n \cdot \nabla u^{n+1}) - u^n \cdot \nabla ^{k+1} u^{n+1})\,\mathrm{d}x\\&\qquad - \int _{{\mathbb {R}}^d} |\nabla ^k u^{n+1}|^2\,\mathrm{d}x - \int _{{\mathbb {R}}^d} \nabla ^k u^{n+1} \cdot \nabla ^k (\nabla W \star \rho ^{n+1})\,\mathrm{d}x\\&\qquad + \int _{{\mathbb {R}}^d}\nabla ^k u^{n+1} \cdot \nabla ^k \int _{{\mathbb {R}}^d}\psi (x-y) (u^n(y) - u^n(x)) \rho ^{n+1}(y)\,\mathrm{d}y\mathrm{d}x\\&\quad =: \sum _{k=1}^5 J_k, \end{aligned} \end{aligned}$$

where $J_1$ and $J_2$ can be estimated as

$$\begin{aligned} J_1 \leqq \Vert \nabla u^n\Vert _{L^\infty }\Vert \nabla ^k u^{n+1}\Vert _{L^2}^2 \leqq M\Vert \nabla ^k u^{n+1}\Vert _{L^2}^2 \end{aligned}$$

and

$$\begin{aligned} \begin{aligned} J_2&\leqq C\left( \Vert \nabla ^k u^n\Vert _{L^2}\Vert \nabla u^{n+1}\Vert _{L^\infty } + \Vert \nabla u^n\Vert _{L^\infty } \Vert \nabla ^k u^{n+1}\Vert _{L^2} \right) \Vert \nabla ^k u^{n+1}\Vert _{L^2} \\&\leqq CM(\Vert \nabla u^{n+1}\Vert _{L^\infty }+ \Vert \nabla ^k u^{n+1}\Vert _{L^2})\Vert \nabla ^k u^{n+1}\Vert _{L^2}. \end{aligned} \end{aligned}$$

For the estimate of $J_4$, we use the fact that *W* is the Coulombian potential to deduce

$$\begin{aligned} \begin{aligned} \left| J_4\right|&= \left| \int _{{\mathbb {R}}^d} |\nabla ^{k} u^{n+1}||\nabla ^2 W \star \nabla ^{k-1}\rho ^{n+1}|\,\mathrm{d}x\right| \leqq \Vert \nabla ^k u^{n+1}\Vert _{L^2}\Vert \nabla ^2 W\Vert _{L^1} \Vert \nabla ^{k-1} \rho ^{n+1}\Vert _{L^2}. \end{aligned} \end{aligned}$$

We next divide $J_5$ into two terms:

$$\begin{aligned} \begin{aligned} J_5&= \sum _{0 \leqq \ell \leqq k} \left( {\begin{array}{c}k\\ \ell \end{array}}\right) \iint _{{\mathbb {R}}^d \times {\mathbb {R}}^d}\nabla ^k u^{n+1}(x) \nabla _x^\ell \psi (x-y) \nabla _x^{k-\ell }(u^n(y) - u^n(x)) \rho ^{n+1}(y)\,\mathrm{d}y\mathrm{d}x\\&= -\sum _{0 \leqq \ell \leqq k-1} \left( {\begin{array}{c}k\\ \ell \end{array}}\right) \iint _{{\mathbb {R}}^d \times {\mathbb {R}}^d}\nabla ^k u^{n+1}(x) \nabla _x^\ell \psi (x-y) \nabla _x^{k-\ell }u^n(x) \rho ^{n+1}(y)\,\mathrm{d}y\mathrm{d}x\\&\quad + \iint _{{\mathbb {R}}^d \times {\mathbb {R}}^d}\nabla ^k u^{n+1}(x) \nabla _x^k \psi (x-y) (u^n(y) - u^n(x)) \rho ^{n+1}(y)\,\mathrm{d}y\mathrm{d}x\\&=: J_5^1 + J_5^2. \end{aligned} \end{aligned}$$

Note that

$$\begin{aligned} \begin{aligned}&\left| \iint _{{\mathbb {R}}^d \times {\mathbb {R}}^d}\nabla ^k u^{n+1}(x) \nabla _x^\ell \psi (x-y) \nabla _x^{k-\ell }u^n(x) \rho ^{n+1}(y)\,\mathrm{d}y\mathrm{d}x\right| \\&\quad = \left| \iint _{{\mathbb {R}}^d \times {\mathbb {R}}^d}\nabla ^k u^{n+1}(x) \nabla _y^\ell \psi (x-y) \nabla _x^{k-\ell }u^n(x) \rho ^{n+1}(y)\,\mathrm{d}y\mathrm{d}x\right| \\&\quad = \left| \iint _{{\mathbb {R}}^d \times {\mathbb {R}}^d}\psi (x-y)\nabla ^k u^{n+1}(x) \nabla _x^{k-\ell }u^n(x) \nabla _y^\ell \rho ^{n+1}(y)\,\mathrm{d}y\mathrm{d}x\right| . \end{aligned} \end{aligned}$$

Thus for $\ell = k-1$ we get

$$\begin{aligned} \begin{aligned}&\left| \iint _{{\mathbb {R}}^d \times {\mathbb {R}}^d}\psi (x-y) \nabla ^k u^{n+1}(x) \nabla u^n(x) \nabla _y^{k-1}\rho ^{n+1}(y)\,\mathrm{d}y\mathrm{d}x\right| \\&\quad \leqq \Vert \nabla u^n\Vert _{L^\infty } \iint _{{\mathbb {R}}^d \times {\mathbb {R}}^d}\psi (x-y)| \nabla ^k u^{n+1}(x)| |\nabla ^{k-1}\rho ^{n+1}(y)|\,\mathrm{d}y\mathrm{d}x\\&\quad \leqq \Vert \nabla u^n\Vert _{L^\infty }\Vert \psi \Vert _{L^1}\Vert \nabla ^k u^{n+1}\Vert _{L^2}\Vert \nabla ^{k-1}\rho ^{n+1}\Vert _{L^2}\\&\quad \leqq CM\Vert \nabla ^k u^{n+1}\Vert _{L^2}\Vert \nabla ^{k-1}\rho ^{n+1}\Vert _{L^2}, \end{aligned} \end{aligned}$$

and for $0 \leqq \ell \leqq k-2$ we obtain

$$\begin{aligned} \begin{aligned}&\left| \iint _{{\mathbb {R}}^d \times {\mathbb {R}}^d}\psi (x-y)\nabla ^k u^{n+1}(x) \nabla _x^{k-\ell }u^n(x) \nabla _y^\ell \rho ^{n+1}(y)\,\mathrm{d}y\mathrm{d}x\right| \\&\quad \leqq \Vert \nabla ^k u^{n+1}\Vert _{L^2}\Vert \nabla ^{k-\ell }u^n\Vert _{L^2}\Vert \psi \Vert _{L^2} \Vert \nabla ^\ell \rho ^{n+1}\Vert _{L^2}\\&\quad \leqq CM\Vert \nabla ^k u^{n+1}\Vert _{L^2}\Vert \nabla ^\ell \rho ^{n+1}\Vert _{L^2}. \end{aligned} \end{aligned}$$

This asserts

$$\begin{aligned} \begin{aligned} J_5^1&\leqq CM\Vert \nabla ^k u^{n+1}\Vert _{L^2}\sum _{0 \leqq \ell \leqq k-2} \left( {\begin{array}{c}k\\ \ell \end{array}}\right) \Vert \nabla ^\ell \rho ^{n+1}\Vert _{L^2} + CM\Vert \nabla ^k u^{n+1}\Vert _{L^2}\Vert \nabla ^{k-1}\rho ^{n+1}\Vert _{L^2}\\&\leqq CM\Vert \nabla ^k u^{n+1}\Vert _{L^2} \Vert \rho ^{n+1}\Vert _{H^{k-1}}. \end{aligned} \end{aligned}$$

Similarly, by integration by parts, we notice that

$$\begin{aligned} \begin{aligned}&\left| \iint _{{\mathbb {R}}^d \times {\mathbb {R}}^d}\nabla ^k u^{n+1}(x) \nabla _x^k \psi (x-y) (u^n(y) - u^n(x)) \rho ^{n+1}(y)\,\mathrm{d}y\mathrm{d}x\right| \\&\quad = \left| \iint _{{\mathbb {R}}^d \times {\mathbb {R}}^d}\nabla ^k u^{n+1}(x) \nabla _y^{k-1} \nabla _x \psi (x-y) (u^n(y) - u^n(x)) \rho ^{n+1}(y)\,\mathrm{d}y\mathrm{d}x\right| \\&\quad = \left| \iint _{{\mathbb {R}}^d \times {\mathbb {R}}^d}\nabla ^k u^{n+1}(x) \nabla _x \psi (x-y) \nabla _y^{k-1}\left( (u^n(y) - u^n(x)) \rho ^{n+1}(y)\right) \,\mathrm{d}y\mathrm{d}x\right| \\&\quad = \left| \sum _{0 \leqq \ell \leqq k-1}\left( {\begin{array}{c}k-1\\ \ell \end{array}}\right) \iint _{{\mathbb {R}}^d \times {\mathbb {R}}^d}\nabla ^k u^{n+1}(x) \nabla _x \psi (x-y) \nabla _y^{k-1-\ell }(u^n(y) \right. \\&\qquad \left. - u^n(x)) \nabla _y^\ell \rho ^{n+1}(y) \,\mathrm{d}y\mathrm{d}x\right| . \end{aligned} \end{aligned}$$

On the other hand, we find that

$$\begin{aligned} \begin{aligned}&\left| \iint _{{\mathbb {R}}^d \times {\mathbb {R}}^d}\nabla ^k u^{n+1}(x) \nabla _x \psi (x-y) (u^n(y) - u^n(x)) \nabla _y^{k-1}\rho ^{n+1}(y) \,\mathrm{d}y\mathrm{d}x\right| \\&\quad \leqq \Vert \nabla u^n\Vert _{L^\infty }\int _{|x-y|\leqq R} |\nabla ^k u^{n+1}(x)|| \nabla _x \psi (x-y)| |x-y| | \nabla _y^{k-1}\rho ^{n+1}(y)| \,\mathrm{d}y\mathrm{d}x\\&\quad \leqq R \Vert \nabla u^n\Vert _{L^\infty } \Vert \psi \Vert _{L^1}\Vert \nabla ^k u^{n+1}\Vert _{L^2} \Vert \nabla ^{k-1} \rho ^{n+1}\Vert _{L^2}\\&\quad \leqq CM\Vert \nabla ^k u^{n+1}\Vert _{L^2} \Vert \nabla ^{k-1} \rho ^{n+1}\Vert _{L^2} \end{aligned} \end{aligned}$$

and

$$\begin{aligned} \begin{aligned}&\left| \iint _{{\mathbb {R}}^d \times {\mathbb {R}}^d}\nabla ^k u^{n+1}(x) \nabla _x \psi (x-y) \nabla _y u^n(y) \nabla _y^{k-2}\rho ^{n+1}(y) \,\mathrm{d}y\mathrm{d}x\right| \\&\quad \leqq \Vert \nabla u^n\Vert _{L^\infty } \iint _{{\mathbb {R}}^d \times {\mathbb {R}}^d}|\nabla ^k u^{n+1}(x)|| \nabla _x \psi (x-y)| |\nabla _y^{k-2}\rho ^{n+1}(y)| \,\mathrm{d}y\mathrm{d}x\\&\quad \leqq \Vert \nabla u^n\Vert _{L^\infty }\Vert \nabla \psi \Vert _{L^1} \Vert \nabla ^k u^{n+1}\Vert _{L^2} \Vert \nabla ^{k-2} \rho ^{n+1}\Vert _{L^2}\\&\quad \leqq CM\Vert \nabla ^k u^{n+1}\Vert _{L^2} \Vert \nabla ^{k-2} \rho ^{n+1}\Vert _{L^2}. \end{aligned} \end{aligned}$$

Moreover, for $0 \leqq \ell \leqq k-3$ we obtain

$$\begin{aligned} \begin{aligned}&\left| \iint _{{\mathbb {R}}^d \times {\mathbb {R}}^d}\nabla ^k u^{n+1}(x) \nabla _x \psi (x-y) \nabla _y^{k-1-\ell }u^n(y) \nabla _y^\ell \rho ^{n+1}(y) \,\mathrm{d}y\mathrm{d}x\right| \\&\quad \leqq \Vert \nabla ^k u^{n+1}\Vert _{L^2} \Vert \nabla \psi \Vert _{L^2} \Vert \nabla ^{k-1-\ell }u^n\Vert _{L^2} \Vert \nabla ^\ell \rho ^{n+1}\Vert _{L^2}\\&\quad \leqq CM\Vert \nabla ^k u^{n+1}\Vert _{L^2} \Vert \nabla ^\ell \rho ^{n+1}\Vert _{L^2}. \end{aligned} \end{aligned}$$

Thus we have

$$\begin{aligned} \begin{aligned} J_5^2&\leqq CM\Vert \nabla ^k u^{n+1}\Vert _{L^2}\sum _{0 \leqq \ell \leqq k-3}\left( {\begin{array}{c}k-1\\ \ell \end{array}}\right) \Vert \nabla ^\ell \rho ^{n+1}\Vert _{L^2} + CM\Vert \nabla ^k u^{n+1}\Vert _{L^2} \Vert \nabla ^{k-2} \rho ^{n+1}\Vert _{H^1}\\&\leqq CM\Vert \nabla ^k u^{n+1}\Vert _{L^2}\Vert \rho ^{n+1}\Vert _{H^{k-1}}, \end{aligned} \end{aligned}$$

and subsequently we get

$$\begin{aligned} J_5 \leqq CM\Vert \nabla ^k u^{n+1}\Vert _{L^2} \Vert \rho ^{n+1}\Vert _{H^{k-1}}. \end{aligned}$$

We finally combine all of the above estimate to have

$$\begin{aligned}&\frac{\mathrm{d}}{\mathrm{d}t}\Vert \nabla ^2 u^{n+1}\Vert _{H^{s-1}} + \Vert \nabla ^2 u^{n+1}\Vert _{H^{s-1}} \leqq CM \Vert \nabla ^2 u^{n+1}\Vert _{H^{s-1}} \\&\quad + CM\Vert \nabla u^{n+1}\Vert _{L^\infty } + CM\Vert \rho ^{n+1}\Vert _{H^s}, \end{aligned}$$

and applying Grönwall’s lemma gives

4.10 $$\begin{aligned} \Vert \nabla ^2 u^{n+1}\Vert _{H^{s-1}} \leqq \Vert \nabla ^2 u_0\Vert _{H^{s-1}} e^{CMt} + E_3(t), \end{aligned}$$

where we used the estimates in **Steps B & C** and $E_3: [0,T_0] \rightarrow [0,\infty )$ is continuous on $[0,T_0]$ satisfying $E_3(t) \rightarrow 0$ as $t \rightarrow 0^+$.

**Step D.-** We now combine (4.5), (4.8), (4.9), and (4.10) to have

4.11 $$\begin{aligned} \begin{aligned}&\Vert \rho ^{n+1}(\cdot ,t)\Vert _{H^s} + \Vert \nabla u^{n+1}(\cdot ,t)\Vert _{L^\infty } + \Vert u^{n+1}(\cdot ,t)\Vert _{L^2(B(0,R))} + \Vert \nabla ^2 u^{n+1}\Vert _{H^{s-1}}\\&\quad \leqq \Vert \rho _0\Vert _{H^s}e^{CMt} + \Vert \nabla u_0\Vert _{L^\infty }e^{(CM - 1)t} + \Vert u_0\Vert _{L^2(B(0,R))} + \Vert \nabla ^2 u_0\Vert _{H^{s-1}} e^{CMt} + E(t) \end{aligned} \end{aligned}$$

for $t \leqq T_0$, where $C>0$ is independent of *n*, and $E: [0,T_0] \rightarrow [0,\infty )$ is continuous on $[0,T_0]$ satisfying $E(t) \rightarrow 0$ as $t \rightarrow 0^+$. On the other hand, the right hand side of (4.11) converges to $\Vert \rho _0\Vert _{H^s} + \Vert u_0\Vert _{L^2(B(0,R))} + \Vert \nabla u_0\Vert _{L^\infty } + \Vert \nabla ^2 u_0\Vert _{H^{s-1}}$ as $t \rightarrow 0^+$ and that is strictly less than *N*. This asserts that there exists $T_* \leqq T_0$ such that

$$\begin{aligned}&\sup _{0 \leqq t \leqq T_*}\Vert \rho ^{n+1}(\cdot ,t)\Vert _{H^s} + \Vert \nabla u^{n+1}(\cdot ,t)\Vert _{L^\infty } + \Vert u^{n+1}(\cdot ,t)\Vert _{L^2(B(0,R))} \\&\quad + \Vert \nabla ^2 u^{n+1}\Vert _{H^{s-1}} \leqq M. \end{aligned}$$

This completes the proof. $\quad \square$

### Proof of Theorem 4.1

We first show the existence of a solution $(\rho ,u) \in {\mathcal {Y}}_{s,R}(T_*)$. Note that $\rho ^{n+1} - \rho ^n$ and $u^{n+1} - u^n$ satisfy

4.12 $$\begin{aligned} \begin{aligned}&\partial _t (\rho ^{n+1} - \rho ^n) + (u^n - u^{n-1})\cdot \nabla \rho ^{n+1} + u^{n-1} \cdot \nabla (\rho ^{n+1} - \rho ^n) \\&\quad + (\rho ^{n+1} - \rho ^n) \nabla \cdot u^n + \rho ^n \nabla \cdot (u^n - u^{n-1}) = 0 \end{aligned} \end{aligned}$$

and

$$\begin{aligned} \begin{aligned}&\partial _t (u^{n+1} - u^n) + (u^n - u^{n-1})\cdot \nabla u^{n+1} + u^{n-1} \cdot \nabla (u^{n+1} - u^n) \\&\quad = - (u^{n+1} - u^n) - \nabla W \star (\rho ^{n+1} - \rho ^n) \\&\qquad + \int _{{\mathbb {R}}^d}\psi (x-y) (u^n(y) - u^{n-1}(y)) \rho ^{n+1}(y)\,\mathrm{d}y\\&\qquad - (u^n(x) - u^{n-1}(x)) \int _{{\mathbb {R}}^d}\psi (x-y) \rho ^{n+1}(y)\,\mathrm{d}y \\&\qquad + \int _{{\mathbb {R}}^d}\psi (x-y)(u^{n-1}(y) - u^{n-1}(x)) (\rho ^{n+1} - \rho ^n)(y)\,\mathrm{d}y, \end{aligned} \end{aligned}$$

respectively. Then multiplying (4.12) by $\rho ^{n+1} - \rho ^n$ and integrating it over ${\mathbb {R}}^d$ gives

4.13 $$\begin{aligned} \Vert (\rho ^{n+1} - \rho ^n)(\cdot ,t)\Vert _{L^2}^2 \leqq C\int _0^t \left( \Vert (\rho ^{n+1} - \rho ^n)(\cdot ,\tau )\Vert _{L^2}^2 + \Vert (u^n - u^{n-1})(\cdot ,\tau )\Vert _{H^1}^2\right) \mathrm{d}\tau ,\nonumber \\ \end{aligned}$$

where $C > 0$ is independent of *n*. On the other hand, for $k=0,1$, we find that

$$\begin{aligned}&\frac{1}{2}\frac{\mathrm{d}}{\mathrm{d}t}\int _{{\mathbb {R}}^d}|\nabla ^k (u^{n+1} - u^n)|^2\,\mathrm{d}x\\&\quad = -\int _{{\mathbb {R}}^d}\nabla ^k (u^{n+1} - u^n) \nabla ^k \left( (u^n - u^{n-1}) \cdot \nabla u^{n+1}\right) \,\mathrm{d}x\\&\qquad -\int _{{\mathbb {R}}^d}\nabla ^k (u^{n+1} - u^n) \nabla ^k \left( u^{n-1} \cdot \nabla (u^{n+1} - u^n)\right) \,\mathrm{d}x\\&\qquad - \int _{{\mathbb {R}}^d}|\nabla ^k (u^{n+1} - u^n)|^2\,\mathrm{d}x -\int _{{\mathbb {R}}^d}\nabla ^k (u^{n+1} - u^n) \nabla ^k (\nabla W \star (\rho ^{n+1} - \rho ^n)(x)) \,dx\\&\qquad +\int _{{\mathbb {R}}^d}\nabla ^k (u^{n+1} - u^n) \nabla _x^k \left( \int _{{\mathbb {R}}^d}\psi (x-y) (u^n(y) - u^{n-1}(y)) \rho ^{n+1}(y)\,\mathrm{d}y\right) \,\mathrm{d}x\\&\qquad -\int _{{\mathbb {R}}^d}\nabla ^k (u^{n+1} - u^n) \nabla _x^k \left( (u^n(x) - u^{n-1}(x)) \int _{{\mathbb {R}}^d}\psi (x-y) \rho ^{n+1}(y)\,\mathrm{d}y\right) \,\mathrm{d}x\\&\qquad +\int _{{\mathbb {R}}^d}\nabla ^k (u^{n+1} - u^n) \nabla _x^k \left( \int _{{\mathbb {R}}^d}\psi (x-y)(u^{n-1}(y) - u^{n-1}(x)) (\rho ^{n+1} - \rho ^n)(y)\,\mathrm{d}y\right) \\&\quad \,\mathrm{d}x =: \sum _{i=1}^7 K_i, \end{aligned}$$

where we easily estimate

$$\begin{aligned} \sum _{i=1}^3 K_i \leqq C\Vert u^{n+1} - u^n\Vert _{H^1}^2+ C \Vert u^n - u^{n-1}\Vert _{H^1}^2. \end{aligned}$$

Here $C>0$ is independent of *n*. We next use the following estimates

$$\begin{aligned} \begin{aligned}&\left| \int _{{\mathbb {R}}^d}(u^{n+1} - u^n)(x) \cdot (\nabla W \star (\rho ^{n+1} - \rho ^n)(x)) \,\mathrm{d}x\right| \\&\quad \leqq C\Vert u^{n+1} - u^n\Vert _{L^2}\Vert \nabla W\Vert _{L^1}\Vert \rho ^{n+1} - \rho ^n\Vert _{L^2}\\&\quad \leqq C\Vert u^{n+1} - u^n\Vert _{L^2}\Vert \rho ^{n+1} - \rho ^n\Vert _{L^2} \end{aligned} \end{aligned}$$

and

$$\begin{aligned}&\left| \int _{{\mathbb {R}}^d}\nabla (u^{n+1} - u^n)(x): (\nabla ^2 W \star (\rho ^{n+1} - \rho ^n)(x)) \,\mathrm{d}x\right| \\&\quad \leqq \Vert \nabla ^2 W\Vert _{L^1}\Vert \nabla (u^{n+1} - u^n)\Vert _{L^2}\Vert \rho ^{n+1} - \rho ^n\Vert _{L^2} \end{aligned}$$

to have $K_4 \leqq C\Vert u^{n+1} - u^n\Vert _{H^1}^2 + C\Vert \rho ^{n+1} - \rho ^n\Vert _{L^2}^2.$ For the rest, if $k=0$, then

$$\begin{aligned} \begin{aligned} K_5&\leqq \Vert u^{n+1} - u^n\Vert _{L^2}\Vert \psi \Vert _{L^2}\Vert u^n - u^{n-1}\Vert _{L^2}\Vert \rho ^{n+1}\Vert _{L^2} \\&\leqq C\Vert u^{n+1} - u^n\Vert _{L^2}^2 + C\Vert u^n - u^{n-1}\Vert _{L^2}^2,\\ K_6&\leqq \Vert u^{n+1} - u^n\Vert _{L^2}\Vert u^n - u^{n-1}\Vert _{L^2}\Vert \psi \Vert _{L^2}\Vert \rho ^{n+1}\Vert _{L^2}\\&\leqq C\Vert u^{n+1} - u^n\Vert _{L^2}^2 + C\Vert u^n - u^{n-1}\Vert _{L^2}^2,\\ K_7&\leqq R\Vert \nabla u^{n-1}\Vert _{L^\infty } \Vert \psi \Vert _{L^1}\Vert u^{n+1} - u^n\Vert _{L^2}\Vert \rho ^{n+1} - \rho ^n\Vert _{L^2} \\&\leqq C\Vert u^{n+1} - u^n\Vert _{L^2}^2 + C\Vert \rho ^{n+1} - \rho ^n\Vert _{L^2}^2. \end{aligned} \end{aligned}$$

On the other hand, if $k=1$, we obtain

$$\begin{aligned} \begin{aligned} K_5&\leqq \Vert \nabla (u^{n+1} - u^n)\Vert _{L^2}\Vert \nabla \psi \Vert _{L^2}\Vert u^n - u^{n-1}\Vert _{L^2}\Vert \rho ^{n+1}\Vert _{L^2} \\&\leqq C\Vert \nabla (u^{n+1} - u^n)\Vert _{L^2} + C\Vert u^n - u^{n-1}\Vert _{L^2}^2,\\ K_6&\leqq \Vert \nabla (u^{n+1} - u^n)\Vert _{L^2} \\&\quad \left( \Vert \nabla (u^n - u^{n-1})\Vert _{L^2}\Vert \psi \Vert _{L^2} + \Vert u^n - u^{n-1}\Vert _{L^2}\Vert \nabla \psi \Vert _{L^2}\right) \Vert \rho ^{n+1}\Vert _{L^2} \\&\leqq C\Vert \nabla (u^{n+1} - u^n)\Vert _{L^2} + C\Vert u^n - u^{n-1}\Vert _{H^1}^2,\\ K_7&\leqq \Vert \nabla (u^{n+1} - u^n)\Vert _{L^2} \\&\quad \left( R\Vert \nabla u^{n-1}\Vert _{L^\infty }\Vert \nabla \psi \Vert _{L^1} + \Vert \psi \Vert _{L^1}\Vert \nabla u^{n-1}\Vert _{L^\infty } \right) \Vert \rho ^{n+1} - \rho ^n\Vert _{L^2}\\&\leqq C\Vert \nabla (u^{n+1} - u^n)\Vert _{L^2} + C\Vert \rho ^{n+1} - \rho ^n\Vert _{L^2}^2. \end{aligned} \end{aligned}$$

We now combine all of the above estimates to have

$$\begin{aligned} \frac{\mathrm{d}}{\mathrm{d}t}\Vert u^{n+1} - u^n\Vert _{H^1}^2 \leqq C\Vert u^{n+1} - u^n\Vert _{H^1}^2+ C \Vert u^n - u^{n-1}\Vert _{H^1}^2 + C\Vert \rho ^{n+1} - \rho ^n\Vert _{L^2}^2, \end{aligned}$$

and subsequently this yields

$$\begin{aligned} \Vert (u^{n+1} - u^n)(\cdot ,t)\Vert _{H^1}^2 \leqq C\int _0^t \left( \Vert (\rho ^{n+1} - \rho ^n)(\cdot ,\tau )\Vert _{L^2}^2 + \Vert (u^n - u^{n-1})(\cdot ,\tau )\Vert _{H^1}^2\right) \,\mathrm{d}\tau , \end{aligned}$$

where $C > 0$ is independent of *n*. This together with (4.13) asserts that $(\rho ^n,u^n)$ is a Cauchy sequence in ${\mathcal {C}}([0,T];L^2({\mathbb {R}}^d)) \times {\mathcal {C}}([0,T];H^1({\mathbb {R}}^d))$. Interpolating this strong convergences with the above uniform-in-*n* bound estimates gives

$$\begin{aligned}&\rho ^n \rightarrow \rho \quad \text{ in } {\mathcal {C}}([0,T_*]; H^{s-1}({\mathbb {R}}^d)), \quad \\&\quad u^n \rightarrow u \quad \text{ in } {\mathcal {C}}([0,T_*]; H^1(B(0,R))) \quad \text{ as } n\rightarrow \infty , \\&\nabla u^n \rightarrow \nabla u \quad \text{ in } {\mathcal {C}}({\mathbb {R}}^d \times [0,T_*]), \quad \text{ and } \quad \\&\quad \nabla ^2 u^n \rightarrow \nabla ^2 u \quad \text{ in } {\mathcal {C}}([0,T_*];H^{s-2}({\mathbb {R}}^d)) \quad \text{ as } n\rightarrow \infty , \end{aligned}$$

due to $s > d/2+1$. We then use a standard functional analytic arguments, see for instances [29, Section 2.1], to have that the limiting functions $\rho$ and *u* satisfy the regularity in Theorem 4.1. We easily show that the limiting functions $\rho$ and *u* are solutions to (4.1) with regularity properties and assumptions of Theorem 1.2.

We finally provide the uniqueness of strong solutions. Let $(\rho ,u)$ and $({\tilde{\rho }}, {{\tilde{u}}})$ be the strong solutions obtained above with the same initial data $(\rho _0, u_0)$. Set $\Delta (t)$ a difference between two strong solutions:

$$\begin{aligned} \Delta (t) := \Vert \rho (\cdot ,t) - {\tilde{\rho }}(\cdot ,t)\Vert _{L^2} + \Vert u(\cdot ,t) - {{\tilde{u}}}(\cdot ,t)\Vert _{H^1}. \end{aligned}$$

Then by using almost the same argument as above, we have

$$\begin{aligned} \Delta (t) \leqq C\int _0^t \Delta (s)\,\mathrm{d}s \quad \text{ with } \quad \Delta (0) = 0. \end{aligned}$$

This concludes that $\Delta (t) \equiv 0$ on $[0,T_*]$ and completes the proof.

## Appendix A. Well-Posedness of the Particle System

In this appendix, we study the global existence and uniqueness of classical solutions to the particle system (1.1)–(1.2).

Let us first consider the case with singular interaction potentials with $d\geqq 2$. In this case, we can use the repulsive effect from the interaction forces, and this also enables us to have the uniqueness of solutions.

### Theorem A.1

Let $d \geqq 2$. Suppose that $\widetilde{W}$ is of the form (2.10) or (2.11) and the confinement potential *V* satisfies either $V \rightarrow +\infty$ as $|x| \rightarrow \infty$ or $\nabla _x V$ has linear growth as $|x| \rightarrow \infty$. If the initial data $x_0$ satisfy

$$\begin{aligned} \min _{1 \leqq i\ne j \leqq N}|x_{i0} - x_{j0}| > 0. \end{aligned}$$

Then there exists a unique global smooth solution to the system (1.1)–(1.2) with $\widetilde{W}$ instead of *W* satisfying

$$\begin{aligned} C \geqq \max _{1 \leqq i\ne j \leqq N}|x_i(t) - x_j(t)| \geqq \min _{1 \leqq i\ne j \leqq N}|x_i(t) - x_j(t)| > 0 \end{aligned}$$

for $t \geqq 0$, where $C>0$ is independent of *t*.

### Proof

For the proof, we first introduce the maximal life-span $T_0 = T(x_0)$ of the initial data data $x_0$ as

$$\begin{aligned} T_0 := \sup \left\{ s > 0 : \text{ solution } (x(t), v(t)) \hbox { for the system } (1.1) \hbox { exists up to the time } s \right\} . \end{aligned}$$

Then by the assumption and continuity of solutions, we get $T_0>0$. We now claim that $T_0 = \infty$ and for this it suffices to show that there is no collision between particles for all $t \geqq 0$ and that particles cannot escape to infinity in finite time.

A straightforward computation yields

$$\begin{aligned} \begin{aligned} \frac{1}{2}\frac{\mathrm{d}}{\mathrm{d}t}\sum _{i=1}^N |v_i|^2&= -\gamma \sum _{i=1}^N |v_i|^2 - \sum _{i=1}^N v_i \cdot \nabla _x V(x_i)\\&\quad - \frac{1}{N}\sum _{i\ne j}^N v_i \cdot \nabla _x \widetilde{W}(x_i - x_j) + \frac{1}{N}\sum _{i,j=1}^N \psi (x_i - x_j)(v_j - v_i) \cdot v_i \end{aligned} \end{aligned}$$

for $t \in [0,T_0)$. Note that

$$\begin{aligned} \frac{\mathrm{d}}{\mathrm{d}t} \sum _{i=1}^N V(x_i) = \sum _{i=1}^N v_i \cdot \nabla _x V(x_i) \end{aligned}$$

and

$$\begin{aligned} \frac{1}{2N}\frac{\mathrm{d}}{\mathrm{d}t}\sum _{i\ne j}^N \widetilde{W}(x_i - x_j) = \frac{1}{2N}\sum _{i\ne j}^N \nabla _x \widetilde{W}(x_i - x_j) \cdot (v_i - v_j) = \frac{1}{N}\sum _{i\ne j}^N \nabla _x \widetilde{W}(x_i - x_j) \cdot v_i, \end{aligned}$$

where we used $\nabla \widetilde{W}(-x) = -\nabla \widetilde{W}(x)$. Similarly, we also find

$$\begin{aligned} \frac{1}{N}\sum _{i,j=1}^N \psi (x_i - x_j)(v_j - v_i) \cdot v_i = -\frac{1}{2N}\sum _{i,j=1}^N \psi (x_i - x_j)|v_j - v_i|^2. \end{aligned}$$

Combining all of the above estimates, we obtain

$$\begin{aligned} \frac{\mathrm{d}}{\mathrm{d}t}{\mathcal {F}}^N(x,v) + \gamma \sum _{i=1}^N |v_i|^2 +\frac{1}{2N}\sum _{i,j=1}^N \psi (x_i - x_j)|v_j - v_i|^2 =0 \end{aligned}$$

for $t \in [0,T_0)$, where ${\mathcal {F}}^N(x,v)$ denotes the discrete free energy given by

$$\begin{aligned} {\mathcal {F}}^N(x,v) := \frac{1}{2}\sum _{i=1}^N |v_i|^2 + \sum _{i=1}^N V(x_i) + \frac{1}{2N}\sum _{i\ne j}^N \widetilde{W}(x_i - x_j). \end{aligned}$$

If $d=2$, then we have either

$$\begin{aligned} \frac{1}{2N}\sum _{i\ne j}^N \frac{1}{|x_i - x_j|^\alpha } \leqq {\mathcal {F}}^N(x_0,v_0) \quad \text{ or } \quad -\frac{1}{2N} \sum _{i \ne j} \log |x_i(t) - x_j(t)| \leqq {\mathcal {F}}^N(x_0,v_0), \end{aligned}$$

where $\alpha \in (0,2)$. On the other hand, if $d \geqq 3$, we obtain

$$\begin{aligned} \frac{1}{2N} \sum _{i\ne j} \frac{1}{|x_i(t) - x_j(t)|^\alpha } \leqq {\mathcal {F}}^N(x_0,v_0) \end{aligned}$$

for all $t \in [0,T_0)$, where $\alpha \in (d-2,d)$. Since the right hand side of the above inequality is uniformly bounded in *t*, we conclude $T_0 = \infty$ for the case $d \geqq 2$. An upper bound estimate of the distance between particles is a simple consequence of the uniform-in-time bound estimate of the free energy ${\mathcal {F}}^N$ due to the confinement potential whenever is present. If $V=0$, one can obtain that particles cannot escape to infinity in finite time as soon as $\nabla _x V$ has linear growth as $|x|\rightarrow \infty$. $\quad \square$

### Remark A.1

If the interaction and confinement potentials *W* and *V* are regular enough, that is $\nabla _x W \in \mathcal {W}^{1,\infty }({\mathbb {R}}^d)$ and $\nabla _x V \in \mathcal {W}^{1,\infty }({\mathbb {R}}^d)$, we have global-in-time existence and uniqueness of solutions by the standard Cauchy-Lipschitz theory. Moreover, an uniform-in-time bound of the distance between particles can also obtained due to the confinement potential if $V \rightarrow +\infty$ as $|x| \rightarrow \infty$.

Let us finally comment on the one dimensional case. If $d=1$ and the interaction potential $\widetilde{W}$ is given by (2.11), then we apply Theorem A.1 to get the global unique classical solution and uniform-in-time bound estimate. If *W* is given by the Coulomb potential, that is

A.1 $$\begin{aligned} W'(x) = \frac{1}{2} sgn(x), \quad \text{ where } \quad sgn(x) := \left\{ \begin{array}{ll} \displaystyle \frac{x}{|x|} &{} \text {if } x \ne 0\\ 0 &{} \text {if } x=0 \end{array} \right. . \end{aligned}$$

Thus the interaction force $- W'$ is discontinuous, but bounded. In this sense, it is not so singular compared to the other cases. Since the velocity alignment force is regular, we can use a similar argument as in [50, Proposition 1.2], see also [10, 41], to have the following proposition.

### Proposition A.1

Let $d=1$. For any initial configuration $\mathcal {Z}^N(0)$, there exists at least one global-in-time solution to the system of (1.1) with (A.1) in the sense that $(x_i(t),v_i(t))$ satisfies the integral system:

$$\begin{aligned} \begin{aligned} x_i(t)&= x_i(0) + \int _0^t v_i(s)\,\mathrm{d}s, \quad i=1,\dots , N, \quad t >0,\\ v_i(t)&= v_i(0) - \gamma \int _0^t v_i(s)\,\mathrm{d}s - \int _0^t V'(x_i(s))\,\mathrm{d}s - \frac{1}{N} \sum _{j\ne i}\int _0^t W'(x_i(s) - x_j(s))\,\mathrm{d}s\\&\quad + \frac{1}{N} \sum _{j=1}^N \int _0^t \psi (x_i(s) - x_j(s))(v_j(s) - v_i(s))\,\mathrm{d}s. \end{aligned} \end{aligned}$$

Even though Proposition A.1 does not provide the uniqueness of solutions, it is not necessary for the analysis of mean-field limit or mean-field/small inertia limit from the particle system (1.1) to the pressureless Euler system (1.3) or the aggregation equation (1.4).
